# Working memory load and search efficiency in conventional monitor-based 2D versus 3D virtual settings: analysis of response times and parietal induced alpha activity in a modified Sternberg task

**DOI:** 10.1007/s00221-026-07266-1

**Published:** 2026-03-09

**Authors:** Merle Sagehorn, Marike Johnsdorf, Joanna Kisker, Thomas Gruber, Benjamin Schöne

**Affiliations:** 1 Experimental Psychology I, Institute of Psychology, Osnabrück University, Lise-Meitner-Str. 3, 49076 Osnabrück, Germany; 2 Department of Psychology, Norwegian University of Science and Technology, Trondheim, Norway

**Keywords:** Working memory, VR, Induced oscillations, Realistic conditions, EEG

## Abstract

In psychophysiological research on memory encoding and retrieval, Virtual Reality (VR) allows for meaningful insights into mnemonic processing under realistic conditions, yet its effects on working memory (WM) processing and performance remain inconclusive. The present study investigates how information search processes in WM differ when stimulus material is encoded in a conventional monitor-based context or in a more realistic virtual setting, and whether complex, naturalistic stimulus properties facilitate or challenge WM processing and task performance. Participants performed a modified Sternberg task with everyday objects presented either on a 2D monitor or in photorealistic VR. To investigate the search mechanism and WM load, response times, accuracy, and parietal induced alpha activity during retention were analyzed. Across both modalities, response times, error rates, and parietal alpha activity increased with setsize, consistent with serial WM search up to capacity limits. Reaction times were faster for target than non-target probes, suggesting that complex object stimuli engage familiarity- or priming-based components in WM search, particularly in VR. Critically, 3D presentation yielded faster response times across setsizes and lower WM load, reflected by reduced parietal alpha activity during retention, while accuracy was preserved. Moreover, response times in the 2D condition deviated earlier from linearity at higher setsizes, whereas 3D presentation resulted in a more gradual increase, indicating delayed capacity-related saturation. Together, the behavioral and electrophysiological findings indicate that more realistic presentation in VR supports more efficient utilization of WM capacity, likely by facilitating access to stored representations, without altering the fundamental serial nature of WM search.

**Supplementary Information:**

The online version contains supplementary material available at 10.1007/s00221-026-07266-1.

## Introduction

Human perception and cognition have evolved to operate in a three-dimensional world. Objects in natural environments possess depth, occupy space, and provide multiple sensory and spatial cues that guide perception and interaction. In contrast, many experimental paradigms rely on two-dimensional representations that omit such properties. While these 2D stimuli serve as useful proxies, they lack key physical features such as dimensionality, depth cues, and realistic size, thereby diverging from the conditions under which the brain oftentimes processes information in real life.

Accumulating evidence indicates that these differences in dimensionality have measurable consequences for cognitive processing (e.g., Johnsdorf et al. 2023; Kisker et al. , ; Kisker et al. 2024a, ; Kisker et al. 2025a, , c; Marini et al. 2019; Nastase et al. 2020; Nejati 2021; Sagehorn, Kisker, et al. 2024a, b; Sagehorn et al. 2023; Sagehorn et al. 2024a, b; Snow & Culham 2021). The perception, encoding, and retrieval of three-dimensional objects engage distinct neural and cognitive mechanisms compared to two-dimensional representations. Studies conducted under more realistic virtual reality (VR) conditions have demonstrated dissociable signatures of visual and visuospatial processing for abstract 3D objects, indexed by differential ERP responses within the P1–N2–P3 complex (Kisker et al. ) and concurrent reductions in cognitive load (i.e., induces theta band response; Kisker et al. 2024a, ). These initial processing steps are followed by in-depth conceptual processing and more extensive encoding of semantic 3D stimuli, such as faces and objects, reflected in modulations of late ERP component complexes (i.e., EPN and LPP) that are sensitive to stimulus-specific information (Sagehorn et al. 2023; Sagehorn et al. 2024a, b) and repetition (Johnsdorf et al. 2023). At retrieval, recognition of 3D everyday objects has been shown to be less dependent on familiarity and more on specific recollection relative to 2D stimuli, as reflected by differences in frontal and parietal old/new effects, respectively (i.e., FN400 and LPC; Kisker et al. , , c). Furthermore, attention and cognitive resource allocation appear to be attuned to the demands of three-dimensional virtual environments, as reflected by variations in the induced alpha and theta band responses, suggesting that such contexts elicit processing patterns more closely aligned with those observed in real-world cognition (Kisker et al. 2024a, ; Sagehorn, Kisker, et al. 2024a, b; Schöne et al. 2021). These findings collectively emphasize that the unique processing of 3D virtual stimuli influences memory formation, motivating an examination of their impact on working memory processes.

Acting as an intermediate between encoding and retrieval, working memory (WM) supports the active maintenance and manipulation of information across perceptual and mnemonic phases. Differences in how items are encoded (e.g., through richer spatial and depth cues or more conceptual processing) may alter WM processing characteristics, potentially impacting the recruiting of retrieval mechanisms such as recollection-based recognition. These WM characteristics include the overall maintenance load and the search strategies used to access stored items. Yet, the functional properties of WM under immersive 3D conditions remain insufficiently characterized, and thus how modality-dependent encoding shapes retention and access in short-term memory, ultimately influencing retrieval performance.

Research on WM in VR has so far concentrated primarily on learning outcomes and potential performance benefits, yielding inconsistent results (for review see Checa & Bustillo 2020). In a game-based setting in a desktop and a VR version, WM performance was enhanced for the VR condition, especially for participants with lower WM capacity (Gabana et al. 2017). In a visual spatial task, an objectively decreased WM load based on electrophysiological measures was observed in 3D compared to 2D (Dan & Reiner 2017). In contrast, the comparison of a more demanding and interactive spatial navigation task in 2D *versus* 3D virtual settings showed that the 3D condition required increased allocation of cognitive resources, resulting in higher WM load but still led to better performance (Slobounov et al. 2015). Learning in a multi-task VR simulation was found to be more demanding and to lead to inferior learning outcome than in 2D, measured both on electrophysiological and behavioral levels (Makransky et al. 2019). It is therefore not possible to generalize the extent to which WM processing exhibits distinct functional characteristics in a more natural, but consequently more complex environment, i.e., is more efficient under conditions it is evolutionarily attuned to.

To situate the investigation of WM in immersive environments within a broader cognitive framework, it is necessary to consider how this system operates under conventional monitor-based 2D conditions. As a central system for the temporary maintenance and manipulation of information, WM supports a wide range of everyday cognitive functions. This accessible short-term information storage is the main purpose of the WM system (Baddeley 2010). However, WM capacity is limited, not only in terms of storage duration but also in terms of the amount and complexity of information that can be retained (Alvarez and Cavanagh 2004; N. Cowan 2001). One key mechanism in WM related to these capacity limitations is the information search process, which is typically investigated using the well-established Sternberg task (Sternberg 1966). The typical setup consists of random stimulus sequences, usually numbers (e.g., Jensen and Tesche 2002) or letters (e.g., Zakrzewska and Brzezicka 2014), followed by a probe stimulus that must be classified as either present (match or target) or absent (non-match or non-target) from the preceding set.

Classical findings using this item-search task indicate that response times increase linearly and accuracy decreases with increasing setsize, consistent with a serial comparison process operating on items held in working memory (e.g., Brzezicka et al. 2019; Freunberger et al. 2009; Schon et al. 2009; Sternberg 1966; Tuladhar et al. 2007; Zakrzewska and Brzezicka 2014). Critically, the defining signature of an exhaustive serial search is that the rate of response time increases with setsize (i.e., the slope) is comparable for target and non-target trials, indicating that all items are scanned regardless of whether a match is detected (Corbin and Marquer 2013; Klabes et al. 2021; Sternberg 1966, 1969). Importantly, while slopes are typically parallel, overall response times (intercepts) often differ between target and non-target trials, with target probes commonly yielding faster responses, likely reflecting response biases or decision-stage processes rather than differences in the search mechanism itself (Klabes et al. 2021; Sternberg 1966, 1969). This characteristic pattern has been replicated across numerous stimulus domains, e.g., faces (Tuladhar et al. 2007), images (Brzezicka et al. 2019) and nature scenes (Schon et al. 2009), indicating that the fundamental properties of the working-memory search process generalize beyond symbolic material.

Despite the robustness of this pattern, alternative models of WM access have been proposed (for extensive review, see Corbin and Marquer 2013), including self-terminating search mechanisms (Clifton and Birenbaum 1970; Corballis and Miller 1973; Klatzky and Atkinson 1970; Theios et al. 1973; Van Zandt and Townsend 1993), and familiarity- or priming-based global matching models (Baddeley and Ecob 1973; Donkin and Nosofsky 2012). These accounts emphasize that, in addition to serial comparison processes, memory trace strength and familiarity can influence response efficiency, particularly at the decision stage. How such mechanisms operate under more realistic 3D virtual conditions, however, remains largely unexplored.

To determine the specific functional properties of the WM search mechanism under more realistic virtual conditions in the context of the Sternberg paradigm, both the behavioral performance and electrophysiological measures provide valuable information. At the electrophysiological level, changes in WM load as a function of task difficulty in the Sternberg task under conventional monitor-based 2D conditions were previously investigated using oscillatory responses, particularly in relation to changes in alpha activity at around 10 Hz (Chen et al. 2023; Jensen et al. 2002; Maurer et al. 2015; Tuladhar et al. 2007; Wianda and Ross 2019). Specifically, the parieto-occipital alpha activity (i.e., synchronization) during retention scaled with increasing setsize, reflecting increasing WM load (Jensen et al. 2002; Schack and Klimesch 2002; Tuladhar et al. 2007). The same pattern has also been observed during the retention period in other visual and auditory WM tasks that systematically manipulated WM load (Kawasaki et al. 2010; Marsella et al. 2017). The mechanism underlying this link between alpha activity and WM load is thought to be the active inhibition of areas involved in WM maintenance, preventing further visual input from interfering with items already stored in memory when the capacity limit is reached (Jensen et al. 2002; Klimesch 2012; Klimesch et al. 1999, 2007; Wianda and Ross 2019). The WM search mechanism requires more cognitive resources the more items are to be retained and searched, which ultimately leads to a blockage of the input stream, which is reflected in the parietal alpha synchronization.

The present study aims to investigate to what extent the specific characteristics of search processes in WM differ when stimuli are encoded and maintained in a conventional monitor-based 2D setting compared to a more realistic virtual context, and whether the more complex but also more naturalistic stimulus features facilitate or constrain WM processing. Therefore, the established Sternberg task (Sternberg 1966) is transferred to virtual 3D conditions using everyday objects as stimulus material. To validate the feasibility of the experimental setup and stimulus material under monitor-based 2D conditions, participants perform the task of detecting a probe stimulus (target vs. non-target trials) in a previously shown stimulus sequence of varying length (setsize; 2–5 objects), both in a conventional 2D setup on a PC monitor and in a 3D-360° VR environment via a head-mounted display (HMD).

In the present study, the term more realistic refers to increased perceptual and spatial fidelity rather than unconstrained real-world interaction. Both the 2D monitor-based and 3D virtual conditions were conducted under controlled laboratory settings, with head movements minimized due to EEG recording constraints. The VR condition provided immersive visual features, including stereoscopic depth cues, binocular disparity, and spatial embedding of objects within a three-dimensional scene. Importantly, the VR environment was visually minimal and nondescript: objects were presented on an identical white table in front of a uniform white wall, and no object-specific or semantically meaningful contextual cues were present. In the 2D condition, objects were presented against a uniform background on a monitor in a non-descriptive room. The monitor-based and virtual conditions were not designed to be perceptually identical with respect to low-level visual features such as stimulus size, viewing distance, or visual angle. Rather than isolating dimensionality while strictly equating all perceptual parameters, the present study aimed to contrast a conventional monitor-based working memory task with an immersive VR implementation that includes intrinsic perceptual richness. These perceptual characteristics necessarily differ from those of 2D monitor presentation and were considered part of the experimental manipulation rather than confounding variables. The goal of this approach was to examine how WM retention processes operate under more realistic viewing conditions, as opposed to tightly controlled monitor-based displays. Importantly, neural analyses focused on the retention period, during which the stimuli were no longer visible. Accordingly, perceptual differences between 2D and 3D presentations were accepted as system-level properties of the respective display modalities. While perceptual differences significantly influence encoding, retention-related EEG activity is less directly driven by preceding visual input. Nevertheless, we acknowledge that encoding-related differences may affect subsequent retention processes, and this limitation is addressed in the discussion.

As for the conventional monitor-based 2D condition, we hypothesize to replicate the classic Sternberg findings of increasing response times and decreasing accuracy (i.e., increased error rates) with increasing setsize, indicative of a serial exhaustive search in working memory. In line with prior work, this is expected to manifest as similar setsize-dependent response-time increases for target and non-target trials, even if overall response times differ between trial types (Brzezicka et al. 2019; Freunberger et al. 2009; Klabes et al. 2021; Schon et al. 2009; Sternberg 1966, 1969; Tuladhar et al. 2007; Zakrzewska and Brzezicka 2014). We further expect the increase in cognitive load with increasing setsize to be reflected in an increase in the parietal induced alpha band response (iABR) during the retention period. Considering the previous inconsistencies when comparing WM and learning processes between monitor-based 2D and virtual 3D conditions (Dan and Reiner 2017; Gabana et al. 2017; Makransky et al. 2019; Slobounov et al. 2015), it is difficult to predict the extent to which WM processing will functionally differ under more realistic conditions in VR. The 3D environment could generally increase the WM load due to higher complexity and thus a higher perceptual load (Makransky et al. 2019; Slobounov et al. 2015). This could be reflected in longer response times, lower accuracy, and increased parietal iABR during retention under VR conditions. The WM load could also be reduced due to the more natural allocation of attentional resources and increased stimulus salience (Constant and Liesefeld 2021; Sagehorn et al. 2024a, b; Schöne et al. 2021). This could, in turn, be reflected in a performance advantage and less cognitive demand in VR, i.e., faster response times with stable or higher accuracy and decreased parietal iABR. The potential differences in task performance and WM load between 2D monitor-based and 3D virtual conditions could help to further elucidate under which circumstances cognitive processing benefits from a more natural context or is associated with additional resource requirements.

## Methods

### Participants

For this study, 35 participants were recruited from the student population of Osnabrück University. All participants were screened for psychological and neurological disorders, and regular drug use prior to the experiment. Participants reporting any psychological or neurological disorders, any uncorrected impairment of vision, current medication, or regular drug use were not allowed to participate in the experiment. If vision correction was needed, participants had to use contact lenses because glasses can be uncomfortable to wear for long periods of time under the EEG cap and VR headset. Previous experiences with computer games, VR environments, and recent usage of such media were documented. All participants gave informed written consent and received partial course credit when applicable.

Due to incomplete data acquisition, 4 participants had to be excluded from all analyses. Specifically, one participant's data file did not contain trigger information, and for three participants, only one condition could be successfully recorded due to technical issues. Ultimately, 31 data sets were selected for data analyses (*M*_age_ = 22.1 years, SD_age_ = 2.5 years, Min_age_ = 19, Max_age_ = 27, 24 female, 30 right-handed).

### Stimulus material

**Fig. 1:**
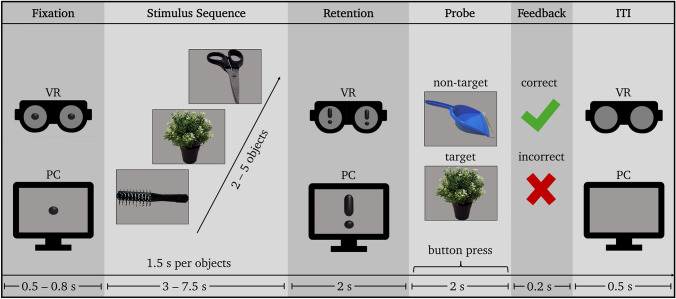
Sternberg trial sequence: 0.5–0.8 s fixation, 3–7.5 s stimulus sequence presentation (1.5 s per object), 2 s delay (retention period), 2 s probe presentation and participant response (retrieval period), 0.2 s feedback, 0.5 s inter-trial-interval (ITI). For illustrative purposes, the fixation dot and exclamation mark is shown in both “eyes” of the VR headset. Only a single fixation dot and exclamation mark was presented to participants within the VR environment during the experiment

**Fig. 2:**
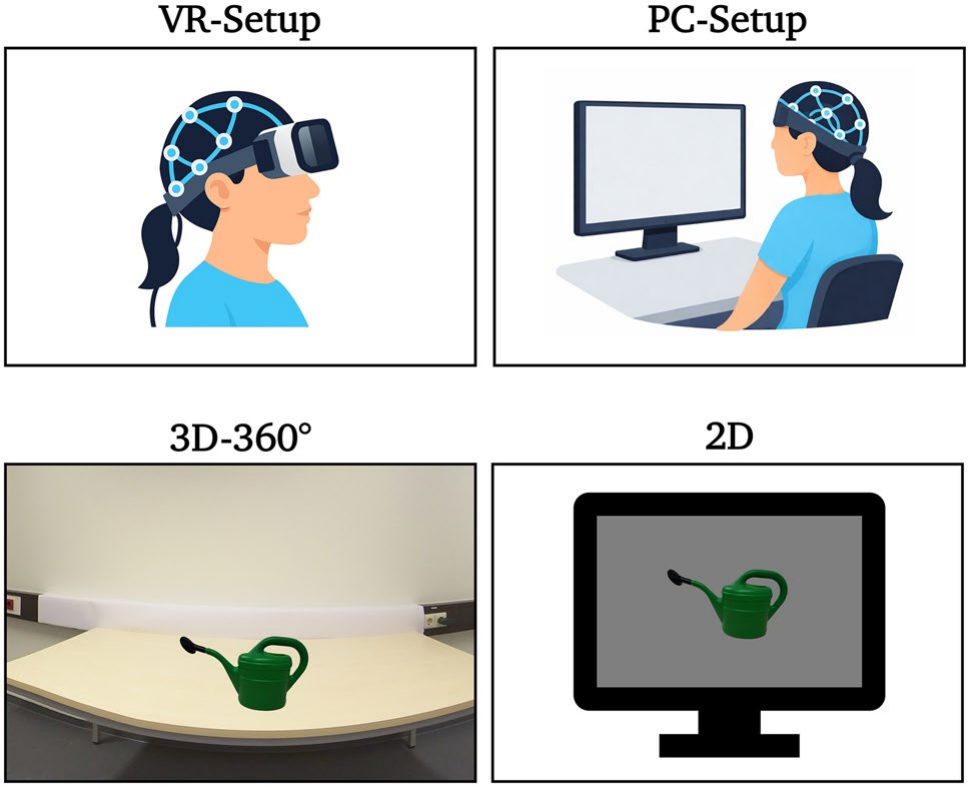
Schematic illustration of the VR- and PC-setups. Examples of object stimuli are given for both modalities

The stimulus material consisted of 60 everyday objects that were chosen from an already existing databank containing both 3D and 2D pictures of the same objects (example stimuli given in Figs. 1 and 2; full list of chosen objects see Table S1, Supplementary Material; for the entire databank see Johnsdorf et al. 2023).

As outlined in the introduction, viewing distance, stimulus size, luminance levels, and viewing angles were not strictly matched between conditions. Rather than isolating dimensionality while strictly equating low-level visual parameters, the present study aimed to contrast a conventional monitor-based WM task with a more ecologically valid immersive VR implementation.

To minimize contextual influences on memory performance, both the 2D and VR environments were intentionally designed to be visually simple and non-descriptive. In the VR condition, objects were presented on a white table in front of a uniform white wall, which remained constant across trials and conditions (see Fig. 2). This environment provided spatial embedding without introducing object-specific or semantically meaningful contextual cues. The 3D pictures were taken with the Insta360 Pro 3D/360° camera at a resolution of 4 K (3840 × 2160 pixels) and viewed as stereoscopic 3D images, thus the stimuli were displayed in their actual size (Fig. 2). The height of the camera lens was standardized for the average sitting human at 112 cm, the table height was 72 cm and the distance from the objects was 62 cm. The objects in the databank ranged in size between 3 and 34 cm, resulting in horizontal and vertical viewing angles from 3° to 34° (Johnsdorf et al. 2023).

In the 2D condition, objects were presented against a uniform background on a monitor placed within an otherwise empty room. Thus, while spatial presentation differed between modalities, contextual information was kept minimal in both conditions. For the conventional monitor-based condition, the pictures were displayed in 2D on a grey background and scaled to a standardized size (Fig. 2), i.e., a maximum 10 cm height of 10 cm and a maximum width of 15 cm width. This resulted in a horizontal viewing angle of 7.5° and vertical viewing angle of 5°.

### Procedure

All participants completed both the PC and the VR condition in a soundproof and electrically shielded room optimized for EEG measurements. The order of the conditions was alternated between participants, i.e., uneven participant numbers completed the PC condition first and even participant numbers completed the VR condition first. Due to the sensitivity of EEG data to artifacts caused by movement, participants were asked to limit their movement and refrain from looking around during the experiment, especially in the VR environment. Participants did not switch location between conditions but were given a five-minute break before completing the second condition during which the EEG signal quality was checked.

For the PC condition, participants were seated in front of a PC monitor (Dell, 24, 1920 × 1200 resolution) with a constant distance of 115 cm to the monitor. The pictures were presented in 2D in the center of the monitor using the video-game engine Unity 5 (Version 2020.3.17f1) for stimulus presentation. For the VR condition, participants were equipped with a VR headset while seated (HTC Vive Pro2, 2448 × 2448 pixel per eye, up to 120° field of vision, 120 Hz refresh rate). The 3D objects were also presented via Unity 5.

The 60 objects were randomly assigned to either the 2D or 3D condition per participant. In both conditions, participants first familiarized themselves with the stimulus material. All 30 objects to be used in the following condition were shown one after the other, and participants were asked to indicate whether or not they recognized the object yet. The answer was given per button-press on a basic USB gamepad with the index fingers. The side of the button press was alternated between participants (even participant numbers pressed right for yes and left for no, odd participant numbers vice versa). The familiarization ended as soon as all objects were recognized and was implemented corresponding to the original study in which the stimulus material, i.e., numbers or letters, were already familiar to the participants.

The participants then performed a modified Sternberg task (Fig. 1). In a total of 124 trials per condition, participants were presented with sequences of two to five objects randomly selected from the stimulus pool of the condition, preceded by a fixation dot (0.5–0.8 s) and followed by an exclamation mark announcing the upcoming query (retention period, 2 s). As soon as the probe picture was displayed (2 s), participants had to answer whether the probe was part of the previous sequence (target trial, response yes) or not (non-target trial, response no). The answer was again given per button-press on a basic USB gamepad with the index fingers, and the participants received feedback on the correctness of their answer after every trial (0.2 s) before the inter-trial-interval (0.5 s). The side of the button press for yes- and no-answers was alternated between the participants in the same way as during the stimulus familiarization. The timepoint of the button-press was recorded and saved to a separate response-time file for each participant by Unity 5 for further analysis. Each condition lasted approximately 25 min, including stimulus familiarization.

As an additional note on the fixation and delay period procedure, the fixation dot was presented at trial onset to standardize initial gaze position but was not continuously displayed during encoding and retention (see similar procedures in Sternberg-type EEG paradigms; Brzezicka et al. 2019; Hwang et al. 2005; Schon et al. 2009). Although small undetected eye movements cannot be entirely ruled out, such micro-movements would be expected to introduce unsystematic noise rather than systematic differences between 2D and 3D conditions. Importantly, the retention interval consistently displayed the same central stimulus across trials and modalities, providing comparable visual input and minimizing differential gaze demands. Given that the reported effects were setsize-dependent and convergent across behavioral and electrophysiological measures, it appears unlikely that subtle gaze deviations account for the observed parietal alpha modulation. With respect to the retention period, an exclamation mark was used to signal the upcoming probe. Given the large number of trials, this ensured that participants knew precisely when to retain the object sequence in memory. During the retention interval, a centrally presented exclamation mark signaled the maintenance phase. Similar placeholder or cue stimuli (e.g., fixation cross, “X”, or textual prompts such as “hold”) are commonly used in Sternberg-type paradigms to structure the trial sequence and minimize temporal uncertainty during retention (e.g., Brzezicka et al. 2019; Maurer et al. 2015; Schon et al. 2009). Although a salient visual stimulus may, in principle, evoke visual processing unrelated to memory maintenance, the stimulus was announced prior to the commencement of the experiment and was therefore anticipated and consistently presented across multiple trials and conditions. Furthermore, baseline correction was conducted within the retention interval, thereby accounting for the potential impact of transient visual responses on the alpha effects (see *Analysis of Oscillatory Responses*). Consequently, the presentation of an exclamation mark during the retention interval is unlikely to account for systematic differences observed between 2 and 3D contexts.

### Behavioral data

For the behavioral data, response times (RTs) for both modalities were recorded for all correct trials and sorted by setsize and trial type (target vs. non-target). RTs were then averaged per participant and condition (8 conditions: PC and VR, setsizes 2–5). For the error rates, trials with incorrect responses were counted separately for each condition. The error rates for each participant thus refer to the number of incorrect trials in each condition.

### EEG recording and preprocessing

Electrophysiological recordings (EEG) were conducted during the entire experiment using 128 electrodes, adhering to the international 10–20 system. The Active-Two amplifier system from BioSemi (Amsterdam, Netherlands) was used with a sampling rate of 512 Hz and a bandwidth (3 dB) of 104 Hz. In addition to the scalp electrodes, a horizontal electrooculogram (hEOG; two electrodes next to the eyes) and a vertical electrooculogram (vEOG; two electrodes above and below the right eye) were recorded. A common mode sense (CMS) and a driven right leg (DRL) electrode were employed as reference and ground electrodes.

The preprocessing of the EEG data from both modalities was conducted using MATLAB (Version R2024a, MathWorks Inc) and EEGLAB (Version 2024.0; Delorme and Makeig 2004) as well as inhouse scripts for wavelet analyses (see below). Initially, the EEG data stream and Unity trigger stream were integrated via the EEGLAB add-on MoBi-Lab (Ojeda et al. 2014). For the continuous data, an automated artifact removal add-on (ASR; default settings; Mullen et al. 2015) was employed for the identification of persistenly noisy channels (ASR default settings: FlatlineCriterion = 5, ChannelCriterion = 0.8, LineNoiseCriterion, 4, Highpass = off, BurstCriterion = off, WindowCriterion = off, BurstRejection = off, Distance = Euclidean). Specifically, channels were flagged if they exhibited prolonged flatlining (> 5 s), poor correlation with neighboring channels, or excessive line noise. Identified channels were subsequently interpolated. Transient high-amplitude bursts were not corrected or rejected within ASR (burst-related criteria were disabled), such that the procedure focused on identifying consistently contaminated channels rather than removing short-lived artifacts. Linear detrending was applied to all channels to eliminate potential slow-wave drifts. For all following preprocessing steps, the EOG channels were excluded.

For the analysis of the retention period, the data was epoched for each condition (i.e., per modality and setsize) as a time window around retention onset of − 500 to 3500 ms with a baseline of − 300 to 0 ms before period onset. The time window size of four seconds was intentionally chosen to include data beyond the delay offset to increase the size of the time samples to the nearest power of two (i.e., 4 s at a sampling rate of 512 Hz results in 2048 time samples). For all data epochs, channels with a signal deviating more than two standard deviations from the mean signal were interpolated. The epochs were high-pass filtered at 0.25 Hz and low-pass filtered at 30 Hz and then re-referenced to an average reference. A 0.25 Hz high-pass filter was applied to remove slow drifts while preserving low-frequency components, consistent with previous studies and our prior published work (Bailey et al. 2023; Delorme 2023; Sagehorn et al. 2023; Sagehorn et al. 2024a, b; Sagehorn, Kisker, et al. 2024a, b). Finally, independent component analysis (ICA; Delorme et al. 2007) was performed. The ICLabel function (v1.4; Pion-Tonachini et al. 2019) was then applied to automatically identify and remove artifacts classified as eye (> 70%), heart and muscle (> 90%) artifacts.

### Analysis of oscillatory responses

To examine spectral changes in oscillatory activity a Morlet wavelet analysis was conducted with a wavelet width of twelve cycles (for detailed documentation see e.g., Bertrand and Pantev 1994; Tallon-Baudry and Bertrand 1999). A total of 39 wavelets covering a frequency range from 2 to 20 Hz was computed resulting in a frequency resolution of 0.5 Hz. This procedure allows for the generation of a time-by-frequency (TF) representation of the data, which offers a dynamic assessment of signal magnitude within each frequency band over time. Time–frequency amplitudes were baseline-corrected on a trial-by-trial basis relative to a pre-retention interval (− 250 to − 50 ms before retention onset) (Luck 2005), i.e., per trial the average amplitude across the baseline time-window was subtracted from every sample. Induced oscillatory activity can be masked within the averaged evoked potential due to variations in latency (Eckhorn et al. 1990). To maintain the signal of interest, i.e., the induced response, we averaged the TF amplitude in the frequency domain, or in other words, across individual frequency transformations for each trial. Furthermore, before conducting the frequency decomposition, the evoked response (or ERP) was subtracted from each trial. This action was performed to enable the analysis of the non-phase-locked components of the signal, which is discussed in greater detail in other works (see Cohen 2014; for similar procedure see e.g., Busch et al. 2006; Gruber et al. 2006). The term “induced frequency band response” thus refers to the non-phase-locked activity obtained after ERP subtraction, i.e., oscillatory activity that is not phase-locked to stimulus onset.

**Fig. 3:**
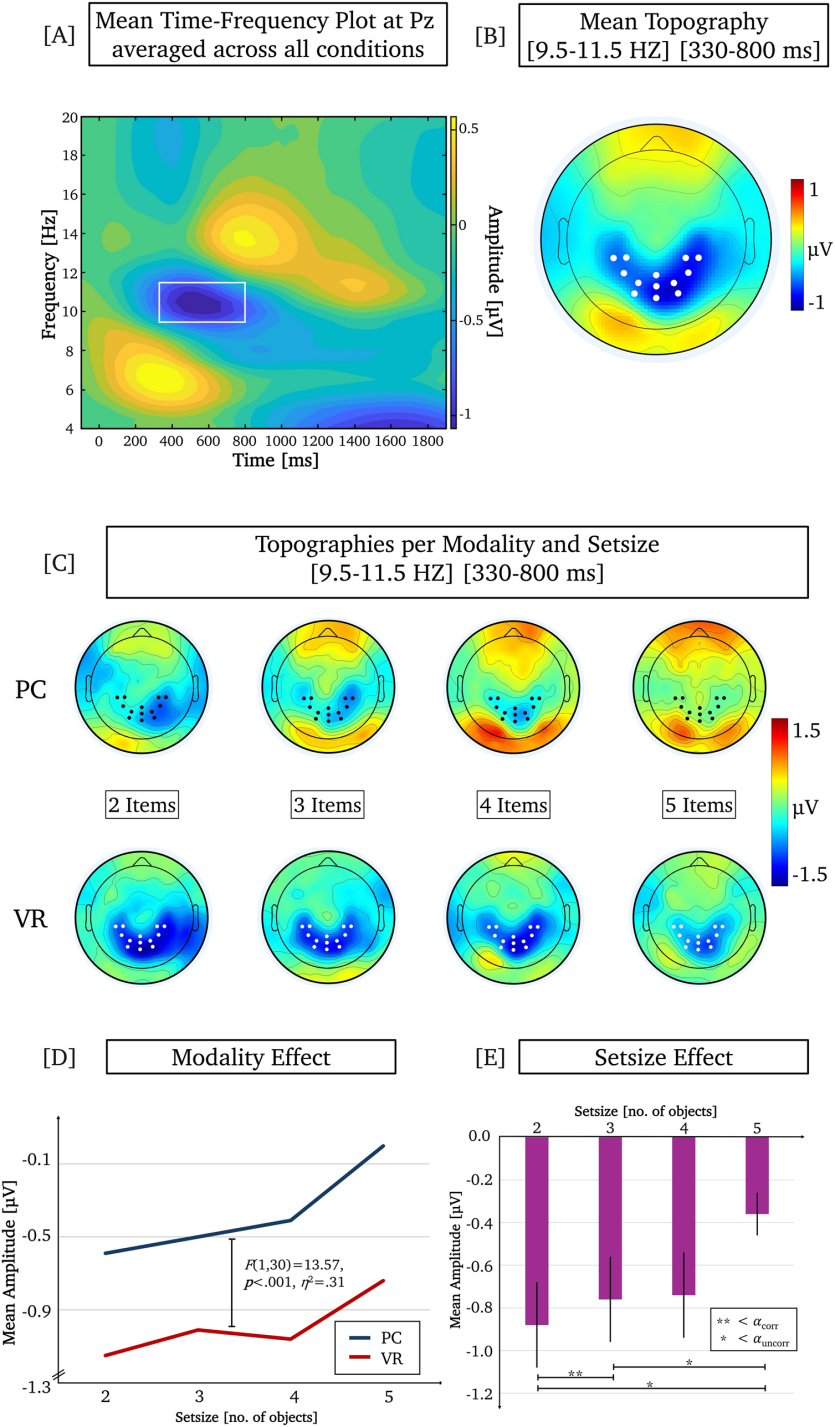
**A** Mean Time–Frequency (TF) plot of the induced oscillatory signal at Pz averaged over all conditions. The iABR frequency range (9.5–11.5 Hz) and time window (330–800 ms) of interest is marked by a white rectangle. **B** Mean topographic distribution of the iABR within the time window of interest, averaged over all conditions. Electrodes selected for analysis are marked by white dots. **C** Topographic distribution of the iABR per modality and setsize within the time window of interest. Electrodes selected for analysis are marked by black or white dots. **D** Mean iABR amplitudes for PC and VR per setsize (modality effect). **E** Mean iABR amplitudes per setsize averaged across modalities (setsize effect). The error bars depict the standard error. Significant differences between the setsizes are highlighted

Based on prior literature, hypotheses were formulated specifically for parietal alpha activity during the retention interval; analyses of other frequency bands were thus not included in the main analyses (see also Discussion Section *Restriction to parietal alpha and consideration of other frequency bands* and Supplementary Material). As conventional in electrophysiological analyses, the selection of the relevant time window for further analyses was informed by previous literature (Chen et al. 2023; Jensen et al. 2002; Maurer et al. 2015; Tuladhar et al. 2007; Wianda and Ross 2019)and based on visual inspection of the grand-mean TF-plot and grand-mean topographic distribution across all conditions (see Fig.3A, B) (for similar procedure see e.g., Gruber et al. 2006; Kisker et al. 2024a, b; Sagehorn et al. 2024a, b). Electrodes were selected for analysis if their amplitude deviated by more than one standard deviation from the mean across all electrodes and if they formed a symmetrical distribution across hemispheres, as no a priori lateralization was assumed (for similar procedure see Kisker et al. , , c). Ultimately, the iABR was analyzed by means of the 9.5–11.5 Hz range at parietal electrodes (i.e., Pz, P1, P2, CP3, CP4, and 7 surrounding electrodes; see e.g., Jensen et al. 2002; Marsella et al. 2017) in the time window from 330 to 800 ms (see Fig. 3) with a baseline set from − 250 to − 50 ms before the retention onset. Since the baseline was set before the retention period and therefore at the end of the encoding period, the differences in WM load depending on the setsize already during stimulus presentation are considered. In other words, the parietal iABR during retention is analyzed in relation to the end of encoding as a function of setsize.

### Statistical analysis

#### Response times and error rates

Response times and error rates (no. of incorrect answers) analyzed using a 2 × 2 × 4 repeated measurements ANOVA (rmANOVA) with the within-subject factors “Modality” (2D vs. 3D), “Target” (target vs. non-target) and “Setsize” (2 items vs. 3 items vs. 4 items vs. 5 items). Whenever necessary, Greenhouse–Geisser-corrected *p*-values are reported. Significant effects of the rmANOVA were complemented by Bonferroni-corrected post hoc *t*-tests.

To determine the duration of each search step in line with the analyses performed in the original Sternberg paradigm (Sternberg 1966), linear regressions were performed for the increase in response time with increasing setsize for target and non-target trials in both modalities. Specifically, the mean response times per setsize (for PC target, PC non-target, VR target, and VR non-target) were set as dependent variables and the number of items (2–5 items) as independent variable, respectively.

#### Parietal iABR

The parietal iABR during the retention period was analyzed using a 2 × 4 rmANOVA with the within-subject factor “Modality” (2D vs. 3D) and “Setsize” (2 items vs. 3 items vs. 4 items vs. 5 items). Whenever necessary, Greenhouse-Geisser-corrected *p*-values are reported. Significant effects of the rmANOVA were complemented by Bonferroni-corrected post hoc *t*-tests.

#### Bayesian statistics

To complement the inferential statistics and to allow for more robust conclusions on possible differences and similarities between conditions (Keysers et al. 2020), all statistical analyses were additionally performed as Bayesian analyses using JASP (JASP Team, 2024), which implements default Bayes factors for ANOVA designs based on Cauchy priors on effect sizes (r = 0.5 for fixed effectes in rmANOVA; r = 0.707 for t-tests; Rouder et al. 2012; Wagenmakers et al. 2018). Specifically, the Bayesian rmANOVAs were performed with the same factors, i.e., a 2 × 2 × 4 Bayesian rmANOVA for the response times and error rates, and a 2 × 4 Bayesian rmANOVA for the parietal iABR. For each factor included in the ANOVA model, the Bayes Factor for the inclusion (*BF*_incl_) of the factor in the model is given (analysis of effects across all models). A *BF*_incl_ > 1 favors the inclusion of the factor in the ANOVA model, while a *BF*_incl_ < 1 favors the exclusion of a factor. For the Bayesian post-hoc *t*-tests and the Bayesian linear regressions, the respective Bayes Factors (H1 compared to H0, *BF*_10_) are reported. A *BF*_10_ > 1 favors the H1, while a *BF*_10_ < 1 favors the H0.

## Results

### Response times

The 2 × 2 × 4 rmANOVA for the response times revealed significant main effects for the factors “Modality” (*F*_Modality_(1, 30) = 4.79, *p* = 0.036, *η*^2^ = 0.14, *BF*_incl_ = 1.4), “Target” (*F*_Target_(1, 30) = 8.85, *p* = 0.006, *η*^2^ = 0.23, *BF*_incl_ = 3.7) and “Setsize” (*F*_*Setsize*_(3, 90) = 69.95, *p* < 0.001, *η*^2^ = 0.70, *BF*_incl_ = 1.8 × 10^+14^). None of the interaction effects were significant (see Table S2, Supplementary Material). The respective descriptive statistics are given in Table S3, Supplementary Material.

**Fig. 4:**
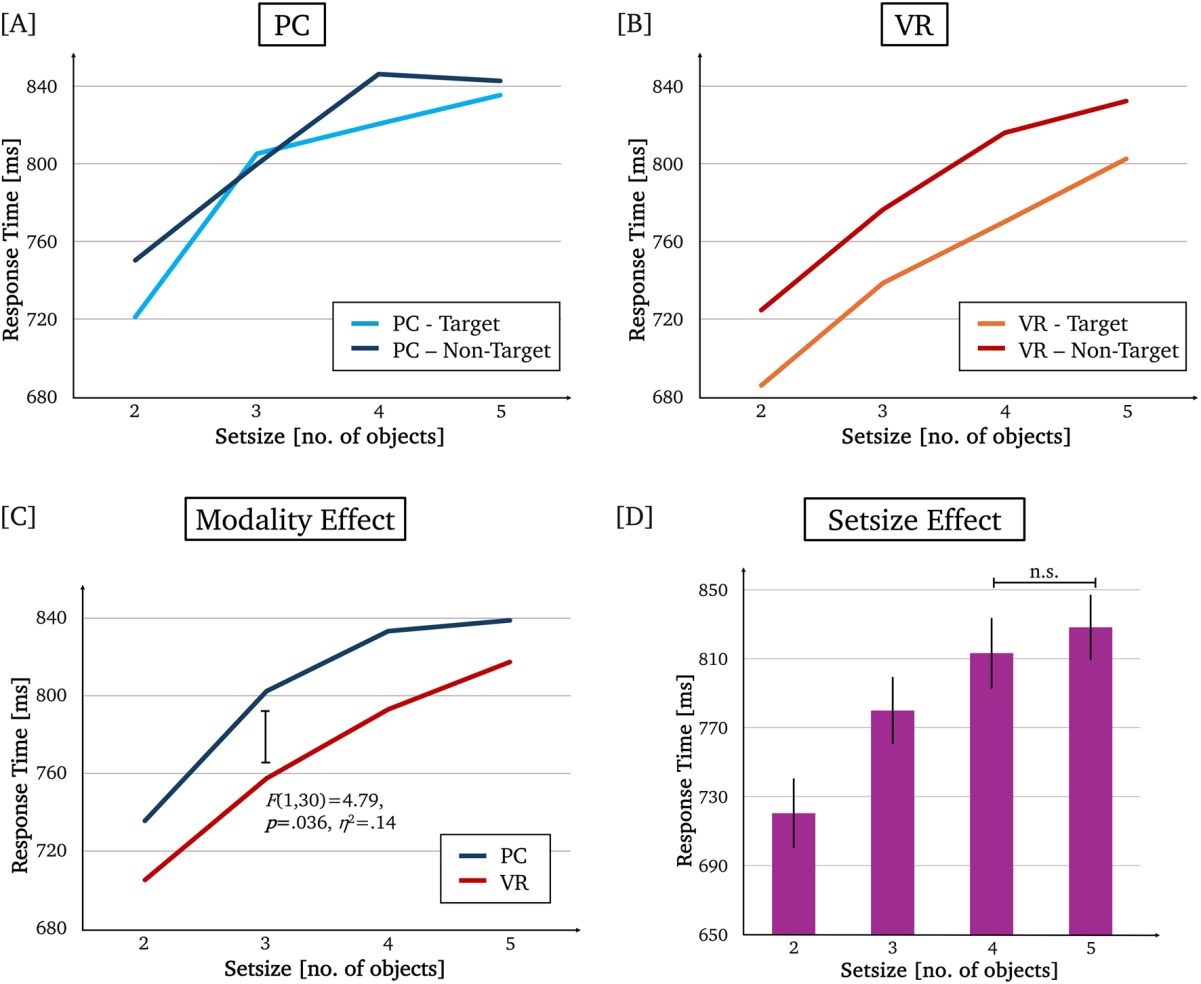
Response time results: Mean response times for **A** PC per setsize for target and non-target-trials, **B** VR per setsize for target and non-target-trials, **C** PC and VR per setsize averaged across target and non-target trials (Modality Effect), and **D** per setsize averaged across modality and target (setsize effect). The error bars depict the standard error. Non-significant differences between the setsizes are highlighted

On average, response times in 3D were significantly faster compared to 2D (*M*_2D_ = 802.56*, M*_3D_ = 768.23; see Fig. 4C) and for targets compared to non-targets (*M*_*T*arget_ = 772.35*, M*_Non-target_ = 798.44; see Fig. 4A and B). Detailed statistics for the pairwise comparisons of response times between setsizes (averaged across modality and target) can be found in Table 1. Larger setsizes elicited significantly longer response times except for 5 compared to 4 items (see Fig. 4D).

**Table 1:** Pairwise comparison of the main factor “setsize” for the response times, error rates and parietal iABR

	*t*(30)	*p* (one-sided*)*	*p* (two-sided)	Cohen´s* d*	*BF*_10_
*Response times*					
2 vs. 3 items	− 9.19	< 0.001**	< 0.001**	− 1.7	1.5 × 10^+12^
2 vs. 4 items	− 10.57	< 0.001**	< 0.001**	− 2.0	2.3 × 10^+19^
2 vs. 5 items	− 12.62	< 0.001**	< 0.001**	− 2.3	7.3 × 10^+27^
3 vs. 4 items	− 4.57	< 0.001**	< 0.001**	− 0.8	480.4
3 vs. 5 items	− 6.00	< 0.001**	< 0.001**	− 1.1	2.3 × 10^+6^
4 vs. 5 items	− 1.65	0.054	0.109	− 0.3	0.6
*Error rates*					
2 vs. 3 items	− 1.97	0.029*	0.058	− 0.35	0.6
2 vs. 4 items	− 3.91	< 0.001**	< 0.001**	− 0.70	735.2
2 vs. 5 items	− 4.31	< 0.001**	< 0.001**	− 0.78	81.8
3 vs. 4 items	− 2.65	0.006**	0.013*	− 0.48	3.0
3 vs. 5 items	− 2.54	0.008*	0.017*	− 0.46	0.8
4 vs. 5 items	1.03	0.156	0.313	0.18	0.1
*Parietal iABR*					
2 vs. 3 items	− 0.94	0.178	0.357	− 0.2	0.2
2 vs. 4 items	− 0.97	0.169	0.338	− 0.2	0.2
2 vs. 5 items	− 3.15	0.002**	0.004**	− 0.6	78.2
3 vs. 4 items	− 0.18	0.430	0.861	0.0	0.1
3 vs. 5 items	− 2.10	0.022*	0.044*	− 0.4	3.2
4 vs. 5 items	− 1.94	0.031*	0.062	− 0.3	2.2

^*^significant for α = 0.05, **significant after Bonferroni-correction (α = 0.008), *BF*_10_ = Bayes Factor for H1 compared to H0. A *BF*_10_ > 1 favors the H1, while a *BF*_10_ < 1 favors the H0

The linear regression for the response times was not significant for either PC target or PC non-target (see Table 2). The linear regression for VR target explains 98.3% of the variance with a duration of each search step of 38.2 ms. The linear regression for VR non-target explains 95.4% of the variance with a duration of each search step of 36.3 ms (see Table 2).

**Table 2:** Linear regressions for the response times [ms] for PC target, PC non-target, VR target, and VR non-target by setsize (no. of objects)

	*df*_*1*_	*df*_*2*_	*F*	*p*	*Intercept*	*b*_1_	*R*^*2*^	*BF*_10_
*Response times*								
PC target	1	2	8.96	0.096	670.0	35.8	0.818	1.6
PC non-target	1	2	12.78	0.070	696.5	32.3	0.865	1.8
VR target	1	2	114.58	0.009*	615.6	38.2	0.983	4.7
VR non-target	1	2	41.13	0.023*	660.1	36.3	0.954	3.0

^*^significant for α = 0.05, *BF*_10_ = Bayes Factor for H1 compared to H0. A *BF*_10_ > 1 favors the H1, while a *BF*_10_ < 1 favors the H0

### Error rates

The 2 × 2 × 4 rmANOVA for the error rates revealed no significant main effect for the factor “Modality” (*F*_Modality_(1, 30) = 0.00, *p* = 1.000, *η*^2^ = 0.00, *BF*_incl_ = 0.1) but for the factors “Target” (*F*_Target_(1, 30) = 6.19, *p* = 0.019, *η*^2^ = 0.17, *BF*_incl_ = 1.9) and “Setsize” (*F*_*Setsize*_(3, 90) = 8.80, *p* < 0.001, *η*^2^ = 0.23, *BF*_incl_ = 56.9), and for the interaction effect “Target*Setsize” (*F*_Target*Setsize_(3, 90) = 2.83, *p* = 0.043, *η*^2^ = 0.09, *BF*_incl_ = 0.8). None of the other interaction effects were significant (see Table S2, Supplementary Material). The respective descriptive statistics are given in Table S3, Supplementary Material.

On average, error rates were higher for targets compared to non-targets (*M*_*T*arget_ = 2.20*, M*_Non-target_ = 1.36). Detailed statistics for the pairwise comparisons of response times between setsizes (averaged across modality and target) can be found in Table 1. Larger setsizes overall elicited higher error rates except for 5 compared to 4 items. The pairwise comparisons between targets and non-targets per setsize can be found in Table S4, Supplementary Material.

### Parietal iABR

The 2 × 4 rmANOVA for the parietal iABR during the retention period revealed significant main effects for the factors “Modality” (*F*_Modality_(1, 30) = 13.57, *p* < 0.001, *η*^2^ = 0.31, *BF*_incl_ = 27.0) and “Setsize” (*F*_*Setsize*_(3, 90) = 3.80, *p* = 0.013, *η*^2^ = 0.11, *BF*_incl_ = 2.2). The interaction was not significant (*F*_*Interaction*_(3, 90) = 0.41, *p* = 0.747, *η*^2^ = 0.01, *BF*_incl_ = 0.2). The respective descriptive statistics are given in Table S2, Supplementary Material. The topographic distribution per modality and setsize is depicted in Fig. 3C.

On average, the parietal iABR was significantly decreased, i.e., stronger negative for 3D than for 2D (*M*_2D_ = − 0.37*, M*_3D_ = − 1.00; see Fig. 3D). Detailed statistics for the pairwise comparison of iABR between setsizes (averaged across modalities) can be found in Table 1 and are illustrated in Fig. 3E. A setsize of 2 items elicited decreased iABR compared to a setsize of 5 items. The comparison of setsizes 3 and 5 and setsizes 4 and 5 did not reach significance after Bonferroni correction. The Bayes factors suggest moderate evidence for a difference between the 3 and 5 items and anecdotal evidence for a difference between the 4 and 5 items. All other comparisons were non-significant (see Table 1).

## Discussion

The present study examined whether and how working memory (WM) search processes differ when stimulus material is encoded under virtual 3D conditions compared to conventional monitor-based 2D presentation, and whether more naturalistic stimulus properties facilitate or challenge WM processing. To this end, a modified Sternberg task using everyday objects was implemented in both a standard monitor-based 2D and a more realistic VR condition.

Behavioral results showed the expected increase in response times and error rates with increasing setsize across both conditions, consistent with classical findings on WM search up to capacity limits. Responses were also faster for target than for non-target probes, indicating that complex object stimuli engage an information search process that is at least partly influenced by familiarity or memory trace strength, in line with extended models of WM search. Notably, in the 2D condition, response times deviated earlier from linearity with increasing setsize, suggesting an earlier onset of capacity-related saturation. In contrast, 3D presentation yielded faster overall responses and a more gradual increase across setsizes, consistent with delayed saturation. Together, these behavioral effects suggest facilitated WM processing under more realistic virtual conditions, without implying a change in the fundamental serial nature of the search process.

At the electrophysiological level, parietal induced alpha activity during retention increased with setsize, replicating its established association with WM load. Across setsizes, alpha activity was reduced under immersive conditions relative to 2D presentation. While this pattern is consistent with lower WM load during maintenance, it may also reflect modality-related differences in attentional engagement or perceptual processing. Taken together, the converging behavioral and electrophysiological findings indicate more efficient utilization of WM resources under more realistic virtual conditions, reflected in reduced WM load during retention and facilitated access to stored information during retrieval.

### Information search mechanism: response times and error rates

The behavioral analysis showed the expected effect of setsize, i.e., an increase in response times and error rates with increasing setsize, independent of trial type and modality, which is the typical effect found in the Sternberg task (e.g., Brzezicka et al. 2019; Freunberger et al. 2009; Schon et al. 2009; Sternberg 1966; Zakrzewska and Brzezicka 2014). Even if a strict linearity of this increase across all setsizes could only be shown under VR conditions, the gradual increase with each additional item to be searched for is consistent with the original result of approx. 40 ms per search step (Sternberg 1966). Based on these results, the search for information in WM with everyday objects as stimulus material still complies with the concept of a serial search mechanism as originally proposed, regardless of the modality in which it is presented.

Moreover, response times differed significantly between target and non-target trials, with participants responding faster to probes that were part of the previously shown stimulus sequence than to probes that were not. Such differences between targets and non-targets are not uncommon in Sternberg-type tasks and have been reported alongside the classic linear setsize effects in numerous studies (Clifton and Birenbaum 1970; Corballis and Miller 1973; Corbin and Marquer 2013; Klabes et al. 2021; Sternberg 1966, 1969). Importantly, within Sternberg’s original framework, the defining characteristic of an exhaustive serial search lies in the similarity of response-time slopes across target and non-target trials, whereas differences in overall response speed are typically attributed to decision-stage processes rather than to the search mechanism itself (Sternberg 1966, 1969). Any deviation from this would have been reflected in a significant interaction between the factors target and setsize, which was not the case in the present study.

One factor likely contributing to the observed target advantage is the nature of the stimulus material. Compared to simple number or letter stimuli, picture stimuli elicit stronger perceptual priming already in 2D, which has been shown to elicit a picture superiority effect in various perceptual WM tasks (Cattaneo et al. 2007; Kinjo and Snodgrass 2000). In general, memory for items presented as pictures reliably exceeds memory for the same items as words in free recall, cued recall, and recognition (Cherry et al. 2008; Ensor et al. 2019; Hockley 2008; Paivio and Csapo 1973; Rajaram 1996; Stenberg 2006). Theoretical accounts for the mechanisms underlying this effect are the perceptual and physical distinctiveness (Ensor et al. 2019; Higdon et al. 2025; Hockley 2008) and moreover, the increased conceptual and semantic richness (Embree et al. 2012; Seifert 1997; Stenberg 2006) of pictures in contrast to simpler stimuli such as words. In fact, the stimulus saliency also has a advantageous effect on WM information processing and consecutive task performance (Constant and Liesefeld 2021).

Even though the interaction between modality and trial type did not reach significance, the target advantage appeared descriptively larger in the more realistic virtual conditions (see Fig. 4A and B) and the Bayes factor suggests anecdotal evidence for the interaction as a contributing factor. Even though this cannot be interpreted as the absence of an effect, further research specifically aimed at this effect is required to determine whether the specific stimulus properties of the 3D material could facilitate the WM search, particularly on target trials.

Nevertheless, this would also be in line with the considerations regarding the picture superiority effect, in this case, in the form of a specific manifestation for VR. In particular, based on the theoretical accounts mentioned above (Embree et al. 2012; Ensor et al. 2019; Higdon et al. 2025; Hockley 2008; Seifert 1997; Stenberg 2006), it could be assumed that the specific perceptual characteristics of more complex, three-dimensional, and immersive stimuli, as well as the in-depth conceptual processing of these (e.g., Sagehorn et al. 2023; Sagehorn et al. 2024a, b), are conducive to memory, especially upon recall. Several studies have shown that presenting stimuli in virtual 3D environments can lead to improved memory performance relative to 2D monitor-based presentation (Schöne et al.^2021^ Kisker, et al.^2025^ ), particularly in spatial (Krokos et al. 2019) and autobiographical memory tasks (Johnsdorf et al. 2025; Schöne et al. 2019). However, this memory advantage cannot be assumed to be universal, as it depends on factors such as presence, interactivity, realism, task demands, and the chosen basis for comparison (Cadet and Chainay 2020; Ceccato et al. 2024; Mizuho et al. 2023; Monaro et al. 2024). Accordingly, it remains an open question whether stimuli presented in virtual 3D settings are perceptually and conceptually distinct from 2D stimuli and therefore may require adapted processing that enables more effective storage already at the level of WM. However, recent studies have shown that processing virtual 3D representations more closely resembles that of real-world representations than that of 2D representations, especially at early visuospatial and visual processing stages (Kisker et al. 2025a; Kisker et al. 2024a, ). This was demonstrated by operationalizing characteristic ERPs and oscillatory responses to index early sensory (P1–N1–P2 complex, evoked theta-band response), attentional processing (induced alpha-band response), and cognitive load (induced theta-band response). Notably, early ERP components (P1–N1–P2) differentiated 2D from 3D and real-life processing, whereas alpha-band activity indicated that attentional demands were not the sole driver of these differences (Kisker et al. 2025). Moreover, results showed that while 2D objects evoked increased cognitive demands, VR and real-life objects elicited largely similar responses, with fewer differences between them than with 2D objects (Kisker et al. 2024a, ). Together, these findings suggest that VR more closely approximates real-world object processing than conventional 2D displays, supporting its use to examine visuospatial and working memory processes in a controlled yet ecologically valid setting.

An unexpected modality-related difference is the attenuated linearity of response times in the 2D condition. Considered in light of the apparent saturation effect at higher setsizes, that is the lack of a significant response time increase between setsizes 4 and 5, this is consistent with a working memory capacity limit (Cowan 2001), and suggests that participants may have reached or exceeded their effective storage capacity at high loads. Under such conditions, serial search assumptions underlying the classic Sternberg linearity no longer strictly apply, as participants may adopt alternative strategies. This might also explain why the error rate is no longer increasing from setsize 4 to 5, as an adjustment to the search mechanism could prevent a further increase in errors. Importantly, this saturation effect was more pronounced in the 2D condition, which likely contributed to the reduced sensitivity of linear regression analyses in this modality. From this perspective, the absence of a significant linear fit in the 2D condition does not indicate a failure to replicate the Sternberg effect but rather reflects earlier or stronger saturation of WM resources. In contrast, the VR condition showed a more gradual increase in response times across setsizes, which may reflect differences in how information is encoded or accessed under immersive presentation, potentially delaying the onset of capacity-related performance plateaus.

Taken together, the considerable overlaps, but also the subtle distinctions in the modality-specific response time profiles, suggest that complex, visually rich stimuli may engage WM search mechanisms that rely at least in part on perceptual priming or familiarity-based access, thus making it easier to decide on the previous presence of a probe rather than its absence. This interpretation aligns with models proposing that more direct access to information in working memory is mediated by memory trace strength rather than by a strictly sequential comparison process (Baddeley and Ecob 1973; Corballis and Miller 1973; Corbin and Marquer 2013; Donkin and Nosofsky 2012). In this framework, direct access refers to a recognition process in which stronger memory traces allow faster and more reliable matching between the probe and stored representations, thereby reducing the need for an exhaustive sequential search. Direct-access models of working memory assume that only a limited number of items can be maintained in a highly accessible state, while other representations remain more weakly activated and require slower retrieval processes (Bocincova et al. 2020; Huynh Cong and Kerzel 2021; Kunda and Ting 2016). This notion closely aligns with embedded-processes models of WM (e.g., Cowan 1999), which distinguish between a limited-capacity focus of attention and a broader set of activated long-term memory representations. Importantly, access to multiple items is subject to capacity constraints and is not fully parallel or cost-free (Tiferet-Dweck et al. 2025). Following this line of argument, it is plausible that the richer perceptual features of 3D stimulus material give rise to stronger memory traces, which may increase the likelihood that items are retained in a highly accessible state during retrieval. This could, in turn, reduce WM demands and contribute to the faster response times observed in the VR condition, potentially delaying the onset of capacity-related saturation effects, without implying a specific or exclusive access architecture.

Apart from the nuanced impact of setsize within and across modalities, the main effect of modality, reflected in generally faster response times without compromising accuracy in the VR condition, suggests a performance advantage under more realistic virtual conditions compared to monitor-based 2D conditions. This pattern is consistent with previous findings in other task domains (e.g., Gabana et al. 2017; Sagehorn et al. 2024a, b) and may indicate more efficient access to information held in WM under immersive VR conditions. Importantly, this modality effect was not trivial in magnitude, as reflected by a large effect size, and converged with independent electrophysiological evidence obtained during the retention interval. Specifically, parietal alpha activity reflecting WM load was systematically reduced in the VR condition, suggesting lower cognitive demands during memory maintenance (discussed in more detail below). Taken together, the correspondence between reduced WM load during retention and faster behavioral responses during retrieval suggests that modality-related differences likely reflect not only perceptual facilitation at probe presentation, but also differences in how stored information is accessed or maintained.

At the same time, the faster response times observed in the VR condition warrant careful interpretation. Because probe stimuli were also presented either in 2D or 3D, perceptual differences at retrieval, such as depth cues, spatial salience, or stimulus discriminability, may have contributed to the behavioral advantage in VR. It should therefore be acknowledged that perceptual and mnemonic factors are intertwined in immersive environments, and that the observed behavioral advantage likely arises from their combined influence rather than from memory-related processes alone. Furthermore, although the VR condition did not provide semantically rich contextual cues, the spatial embedding of objects within a three-dimensional scene may itself function as a form of context and could influence encoding or retention processes, an issue that warrants further investigation.

### Working memory load: parietal iABR

Regarding the parietal iABR, we found that with an increasing number of items to retain in WM, the parietal iABR became less negative, in other words, alpha activity increased with increasing setsize. This indicates that the parietal iABR reflects an active inhibition of areas associated with WM maintenance by impeding additional input from interfering with items already stored in memory when the capacity limit is approached (Jensen et al. 2002; Klimesch et al. 1999; Wianda and Ross 2019). This effect has previously been observed in the Sternberg task (Jensen et al. 2002; Wianda and Ross 2019) and other WM tasks (Marsella et al. 2017), and further supports the link between parietal alpha activity and WM load (Chen et al. 2023). Although the setsize effect for the parietal iABR is not as pronounced across all setsizes as for the response times, the two correspond in showing that WM maintenance and information access become more difficult as the amount of information increases, thus increasing WM load, which in turn contributes to a longer search duration. Apart from the setsize effect, we also observed an effect of modality, i.e., reduced parietal iABR under more realistic virtual compared to monitor-based 2D conditions. The increased alpha activity under monitor-based 2D conditions suggests higher WM load for the task performed in a 2D setting compared to a 3D environment.

This observed modality-related reduction in load in the virtual setting aligns with previous research suggesting WM or cognitive load to be reduced for tasks performed in VR (Dan and Reiner 2017; Sagehorn et al. , b). Conceptually, several interrelated mechanisms may be responsible for this effect. One possibility is a more efficient allocation of attentional resources under VR conditions, driven by increased stimulus salience and perceptual organization that more closely resemble real-world viewing conditions (Constant and Liesefeld 2021; Sagehorn et al. 2024a, b; Schöne et al. 2021). Importantly, this reduction in WM load occurred despite the 3D stimuli being more complex in terms of visual features such as size, spatial layout, and depth cues. Rather than increasing processing demands, these features may thus support more effective encoding by providing additional spatial and perceptual cues that facilitate individuation and maintenance in WM. In this sense, visual complexity does not necessarily translate into higher cognitive load if it is structured in a way that supports task-relevant encoding. Ultimately, it is crucial not only to compare monitor-based 2D and virtual 3D conditions, but also to include a comparison with real-world representation to determine which conditions offer a better approximation. Such trifold comparisons are still rare, but first findings show that the cognitive load involved in object processing under virtual 3D conditions is reduced compared to monitor-based 2D conditions but, importantly, is comparable to that involved in processing real objects (Kisker et al. 2024a, ).

While the observed setsize effects support a canonical interpretation of parietal alpha as a marker of WM load, the modality-related alpha differences merit additional consideration. Parietal alpha activity during retention is well-established as a marker of WM load, reflecting active inhibition of task-irrelevant input (Jensen et al. 2002; Klimesch 2012; Klimesch et al. 1999, 2007; Tuladhar et al. 2007; Wianda and Ross 2019). In the present study, alpha increased with setsize, consistent with this load-dependent mechanism. The modality effect, with reduced alpha in the VR compared to the monitor-based condition, is interpreted as indicating lower WM load, potentially facilitated by stronger memory trace formation and optimized attentional allocation due to enhanced perceptual features of the immersive VR stimuli. At the same time, we acknowledge that these modality-related differences may also reflect sustained attentional engagement or perceptual processing inherent to the virtual environment. Alpha band activity is also understood as a mechanism for suppressing the processing of irrelevant information, which allows alpha suppression to be interpreted as a reverse index of increased attentional engagement (Başar et al. 2000; Klimesch et al. 1998; Köster and Gruber 2022). As such, alpha band desynchronization has generally been linked to enhanced sensory, attentional, and emotional processing (e.g., Codispoti et al. 2023; Diepen et al. 2019; Woodman et al. 2022). Therefore, while setsize effects support the canonical link between parietal alpha and WM load, modality effects potentially reflect a combination of working memory, attentional, and perceptual factors.

One way in which the distinct perceptual and attentional factors influencing processing of virtual 3D stimuli may translate into reduced working memory load is through mechanisms described by global access and familiarity-based models of WM (Corbin and Marquer 2013; Donkin and Nosofsky 2012). Within this framework, more realistic presentation in VR may support the formation of stronger memory traces, thereby facilitating more efficient access to stored information. Memory trace strength has traditionally been linked to factors such as repetition (Baddeley and Ecob 1973) or serial position effects (Corballis and Miller 1973), but it may likewise be modulated by stimulus characteristics that enhance perceptual distinctiveness and spatial embedding. In the present context, features such as color, three-dimensional structure, and depth cues may contribute to more robust encoding, increasing the accessibility of representations during maintenance and retrieval without necessarily increasing overall cognitive load.

Support for this interpretation also comes from converging evidence demonstrating that stimuli presented in real proportions engage distinct perceptual processing pathways (Kisker et al. 2025a; Kisker et al. 2024a, b; Sagehorn et al. 2023; Sagehorn et al. 2024a, b), more elaborate encoding mechanisms (Johnsdorf et al. 2023) and modality-specific retrieval mechanisms (Kisker et al. , , c). The modality therefore influences cognitive processing as early as the stimulus presentation phase and may give rise to differences in how information is represented in working memory when 2D versus 3D objects are encoded. In the present study, WM representations themselves were not directly operationalized or measured. Accordingly, differences in representational strength or structure are proposed here as a theoretical interpretation rather than an empirically tested mechanism, informed by prior literature on cognitive and mnemonic processing in VR (Johnsdorf et al. 2023; Kisker et al. ; Kisker et al. 2024a, b; Kisker et al. 2025a, , c; Sagehorn et al. 2023; Sagehorn et al. 2024a, b). Assuming a stronger priming effect of the 3D stimulus material at this point, the trace strength of 3D objects could be stronger than for 2D objects. Consequently, the set of items maintained in WM, which comprises multiple representations that may differ in trace strength, could differ systematically between 2D and 3D conditions, potentially requiring different search or maintenance dynamics. From this perspective, the physiological and behavioral effects observed in the present study are likely shaped by the joint contribution of enhanced perceptual encoding, more efficient WM maintenance, and facilitated access during retrieval, rather than by a single isolated mechanism. Although the present study cannot disentangle these stages, previous research has distinguished the contributions of encoding quality, maintenance-related interference, and retrieval dynamics to working memory performance (e.g., Oberauer and Lin 2023; Ren et al. 2025; Tabi et al. 2021). For example, processing-stage-specific manipulations, such as distractor timing, show that encoding-stage distraction and maintenance-stage distraction have distinct signatures: longer exposure to encoding-stage distraction can be overcome by better consolidation, whereas post-consolidation distractors remain disruptive regardless of initial encoding quality (Ren et al. 2025). Moreover, information filtering demands at encoding, during maintenance, and via retrieval cues each impair performance, yet individual differences in these abilities are uncorrelated, arguing for distinct processes at each phase (Tabi et al. 2021). Interference models further suggest that errors and serial position effects arise from interference in context–item bindings and output, rather than from passive decay over time (Oberauer and Lin 2023), highlighting that maintenance and retrieval processes can have specific, measurable contributions to performance patterns. These findings illustrate that encoding, maintenance, and retrieval can be experimentally separated, with distinct behavioral and neural signatures, and suggest that future VR-based WM studies could isolate these stages to examine how immersive environments modulate each component of memory processing.

Notably, the modality-related patterns observed at both the physiology and behavioral level are consistent in direction: reduced WM load inferred from parietal iABR during retention coincides with faster response times during retrieval under immersive VR conditions. This parallel pattern is suggestive of a prospective link between reduced WM load and faster response times in more realistic conditions, but the relationship remains speculative and should be interpreted cautiously.

While the present findings suggest that more realistic stimulus presentation in VR can support WM processing, they must be reconciled with a body of work reporting null or opposing effects. In contrast to the present results, other studies investigating the impact of task implementation in VR on WM load and performance outcome have reported increased cognitive demands (Slobounov et al. 2015) or poorer performance in VR (Makransky et al. 2019). These seemingly divergent findings likely reflect differences in task demands, the degree and type of immersion, and the extent to which VR introduces additional processing requirements that are not directly relevant to the target cognitive process (e.g., navigation, motor coordination, or divided attention). From a cognitive load perspective, overall load reflects the interaction between intrinsic task demands, extraneous processing requirements, and task-supporting factors (Kirschner et al. 2011). Accordingly, immersive environments may increase cognitive load when they add task-irrelevant complexity but may reduce load when additional perceptual or spatial cues support task-relevant processing. Consistent with this view, depth cues have been shown to facilitate visuo-spatial processing (Dan and Reiner 2017; Kisker et al. 2024 ), whereas complex navigational demands may impose additional load (Slobounov et al. 2015). A recent review further suggests that 3D environments can enhance learning and engagement, particularly in spatial tasks, while their effects on cognitive load depend on task complexity and resulting demands (Khan et al. 2025). In the present study, VR primarily served to manipulate realism via stimulus dimensionality, photorealism, and realistic size, while keeping task demands comparable. Under these conditions, more realistic presentation in VR appears to support perceptually driven WM processes rather than imposing additional extraneous load. Against this background, the present findings suggest that immersive VR can support WM processing when its perceptual affordances align with task-relevant encoding and maintenance demands. Taken together, VR does not uniformly increase or decrease cognitive demands. Instead, its effects on WM depend on how immersive features interact with task-relevant encoding, maintenance, and retrieval processes, an issue that warrants systematic investigation in future work.

### Limitations

#### Perceptual differences and interpretation of modality effects

A central limitation of the present study concerns the comparability of low-level perceptual features between the 2D monitor-based and immersive VR conditions. Viewing distance, retinal stimulus size, luminance, contrast, and depth cues were not strictly matched across modalities. These factors are known to influence visual processing and may affect both cognitive processing during stimulus encoding and behavioral performance, including response times.

Importantly, these differences were not treated as unintended confounds but as intrinsic properties of immersive VR. Rather than isolating dimensionality under tightly controlled perceptual conditions, the present study aimed to contrast a conventional monitor-based working memory task with a more ecologically valid VR implementation. Perceptual richness, spatial embedding, and depth information are therefore considered part of the system-level manipulation inherent to immersive environments.

Electrophysiological analyses focused on the retention period, during which no visual input was present. While perceptual differences between 2D and VR presentation significantly influence encoding-related activity and subsequently behavioral responses (e.g., Sagehorn et al. 2024a, b; Sagehorn, Kisker, et al. 2024a, ), their direct contribution during retention is reduced. Parietal alpha activity during this phase has been widely linked to WM load via active inhibition of areas involved in WM maintenance (Jensen et al. 2002; Klimesch 2012; Wianda and Ross 2019), rather than exclusively with ongoing sensory processing. Nonetheless, encoding-related differences due to perceptual characteristics of the stimulus material may propagate into subsequent retention processes and indirectly shape response speed and neural dynamics, and should therefore be considered when the interpreting modality-dependent effects.

Within these constraints, the present findings suggest that WM retention dynamics differ between monitor-based 2D and virtual 3D environments. The observed modulation of parietal alpha activity indicates that neural mechanisms supporting WM maintenance are sensitive to the broader perceptual and spatial context in which information is encoded. Parallel differences in response times further suggest that immersive environments modulate both behavioral efficiency and neural signatures of WM. These effects are unlikely to reflect simple sensory differences alone but may instead index changes in attentional demands or representational strategies induced by immersive 3D environments. Future research could further disentangle perceptual and cognitive contributions by parametrically manipulating visual features such as stimulus size, luminance, or viewing distance within VR, or by closely matching retinal input across 2D and 3D displays. Combining such controlled approaches with ecologically valid task designs may help clarify which aspects of immersive environments drive changes in working memory processing. From an applied perspective, these findings underscore that neural markers of working memory are modality-dependent, highlighting the importance of establishing modality-specific baselines when using VR in cognitive assessment or training contexts (see *Implications for Applied and Clinical Contexts*).

#### Restriction to parietal alpha and consideration of other frequency bands

The present study focused on parietal alpha activity as the primary neural correlate of working memory load. This decision was guided by strong theoretical and empirical evidence linking parietal alpha modulation during retention to working memory maintenance via inhibition of task-irrelevant sensory input (Jensen et al. 2002; Klimesch 2012; Schack and Klimesch 2002; Tuladhar et al. 2007; Wianda and Ross 2019). While time–frequency representations revealed activity across wide-spread electrode sites and multiple frequency bands, the main analyses were intentionally restricted to a priori defined parietal alpha dynamics to maintain a focused, hypothesis-driven approach.

Based on the valuable suggestion of one reviewer, the apparent alpha band modulations at posterior electrodes (see Fig. 3B and C) were additionally examined within the same time window as the parietal iABR. This exploratory analysis revealed a pattern consistent with the parietal alpha results (see Supplementary Material, S5, S8–S10), although it remains unclear whether this reflects independent activity or is dependent on the same underlying dipole, thus precluding definitive conclusions about separate activity.

Although frontal theta activity has been robustly associated with executive control and working memory in previous work (Castro-Meneses et al. 2020; Dan and Reiner 2017; Parto-Dezfouli et al. 2025; Puszta 2022), additional analyses in the present dataset did not reveal systematic theta modulation as a function of working memory load or presentation modality. Moreover, theta topographies did not exhibit the canonical mid-frontal distribution typically reported in the literature, further limiting the interpretability of these effects (see Supplementary Material, S6, S8–S10).

Similarly, beta-band activity was not included in the original analyses due to the absence of a priori hypotheses linking beta oscillations to the specific research questions addressed here. Exploratory analyses revealed a posterior beta modulation as a function of working memory load but no differences between display modalities (see Supplementary Material, S7–S10). Notably, this effect emerged temporally after the parietal alpha modulation and partially overlapped with alpha-band synchronization, suggesting that it may reflect a secondary or downstream process rather than an independent mechanism of working memory maintenance.

Taken together, these observations support the decision to retain parietal alpha as the primary focus of the present study while acknowledging the presence and potential relevance of additional frequency-specific dynamics. Future studies may explicitly target theta, alpha, and beta activity or adopt multimodal analytic approaches to further elucidate how immersive environments shape distributed working memory networks.

### Implications for applied and clinical contexts

Beyond working memory, the present findings have broader implications for the study of cognition under immersive conditions. Three-dimensional and immersive stimulus presentation has been shown to modulate neural and behavioral responses in other domains, including fear processing, threat perception, and defensive behavior, where spatial realism and depth cues amplify salience, proximity, and emotional engagement (Cong et al. 2025; Kisker et al. 2021; Rosén et al. 2019). Across studies, immersive 3D and multisensory presentations reliably increase arousal, presence, and proximal defensive responses, and can impair concurrent cognition, particularly when threat is near or dynamically approaching (Cong et al. 2025; Rosén et al. 2019). Effects on learned or conditioned fear and on stress reduction are more nuanced and not always enhanced by immersion (Rosén et al. 2019). Extending the present paradigm to such domains could help determine whether VR-based stimuli elicit more robust or ecologically valid neural signatures than conventional 2D displays, particularly for processes that are inherently spatial or embodied in nature (e.g., Serrratos Hernandez et al. 2023).

At the same time, these results carry important implications for applied and clinical contexts (Garrett et al. 2018; Park et al. 2019; Voinescu et al. 2021). While VR may enhance ecological validity and, under certain task conditions, reduce working memory load or cognitive fatigue, it also establishes modality-specific physiological baselines. Differences in neural markers such as parietal alpha activity may therefore reflect properties of the display medium itself rather than changes in cognitive status. This is particularly relevant for neuropsychological assessment, where results obtained in immersive VR environments cannot be directly compared to normative data acquired under conventional 2D testing conditions without accounting for modality-related offsets (Garrett et al. 2018). More generally, these findings highlight the need for careful calibration and validation when adopting VR-based paradigms in clinical, educational, or applied settings (Garrett et al. 2018; Park et al. 2019; Voinescu et al. 2021), ensuring that observed benefits in realism or engagement are appropriately interpreted in relation to underlying cognitive and neural processes.

### Conclusions

In the present study, the Sternberg task as a well-established WM paradigm was successfully transferred to more realistic conditions by using everyday objects as complex stimuli in a standard monitor-based 2D compared to a 3D virtual setting. Behaviorally, response times increased with setsize in both modalities, consistent with a serial search mechanism in WM at least up to the capacity limit. In addition, responses were faster for target than for non-target probes, indicating that the use of complex object stimuli engages an information search process that is at least partly influenced by familiarity or memory trace strength, in line with prior extensions of the Sternberg framework.

Crucially, behavioral performance differed between modalities. In the 2D condition, response times showed an earlier deviation from linearity with increasing setsize, suggesting an earlier onset of capacity-related saturation effects. In contrast, the VR condition was characterized by faster overall response times and a more gradual increase across setsizes, indicating delayed saturation under immersive presentation. This performance advantage in VR suggests that immersive 3D presentation facilitates access to information retained in WM, without implying a change in the fundamental serial nature of the search process.

At the electrophysiological level, parietal alpha activity during retention increased with setsize, replicating its established association with WM load. Across setsizes, alpha activity was reduced in the VR condition relative to 2D presentation. This modality-related reduction in WM load parallels the behavioral advantage observed in VR. While this effect is consistent with reduced WM load during maintenance under VR conditions, it may also reflect modality-related differences in attentional engagement or perceptual processing inherent to VR environments.

Taken together, the behavioral and electrophysiological findings converge in suggesting that immersive 3D presentation supports more efficient utilization of available working memory resources. Faster response times, delayed saturation effects, and reduced parietal alpha activity jointly point to lower effective cognitive demands under more realistic virtual conditions. One plausible integrative account is that the distinct encoding of the 3D virtual stimuli gives rise to stronger or more accessible memory traces in working memory. Such strengthened representations would facilitate access during retrieval, reduce effective search demands, and delay the onset of capacity-related performance limitations. Importantly, this interpretation does not imply a change in the fundamental serial nature of the search process, but rather suggests that modality-dependent encoding characteristics influence how efficiently stored information can be accessed within existing working memory constraints. Future work that directly operationalizes representational strength or format will be necessary to further specify these mechanisms.

Further implementation of different WM paradigms in more realistic experimental setups as opposed to monitor-based settings will help to gain further insights into real-life relevant memory processing and furthermore advance the practical application of VR in psychological WM training and assessment.

## Supplementary Information

Below is the link to the electronic supplementary material.

Supplementary file1.

## Data Availability

The dataset including aggregated behavioral and EEG data per participant and SPSS Syntax for the statistical analyses performed in this study can be found at OSF (https://osf.io/3uag5/overview?view_only=190e89b1bf6a46b3aa2d00b33a2a66e1). The raw EEG data generated during the study and preprocessed and analyzed for the study are available upon reasonable request to the author.
