# Supplementary material for: Working memory load and search efficiency in conventional monitor-based 2D versus 3D virtual settings: analysis of response times and parietal induced alpha activity in a modified Sternberg task
Source: Exp Brain Res. 2026 Mar 9;244(4):62. doi: 10.1007/s00221-026-07266-1 (PMC12971832; doi:10.1007/s00221-026-07266-1)
Supplement: Supplementary file 1 — Supplementary file1. [file 221_2026_7266_MOESM1_ESM.docx]

**Supplementary Material**

**Table S1**

*List of objects used as stimuli*

| **1 – Thermos Mug** | **2 – Tea Tin** | **3 – Salt/Pepper Mill** | **4 – Duct Tape** |
| --- | --- | --- | --- |
| 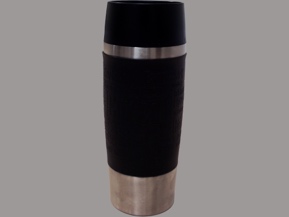 | 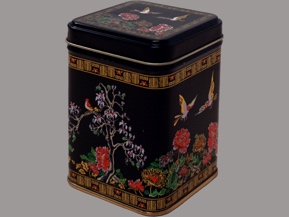 | 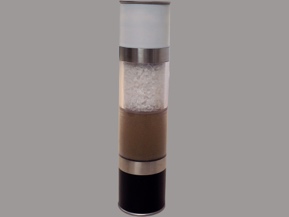 | 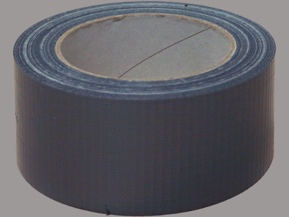 |
| **5 – Tool Box** | **6 – Scissors** | **7 – Headphones** | **8 – Trophy** |
| 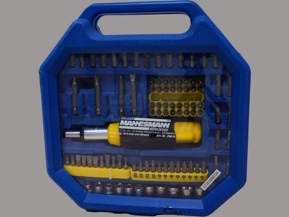 | 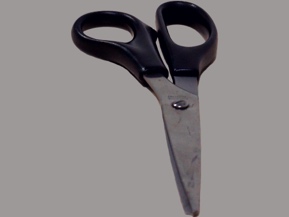 | 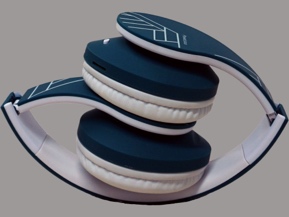 | 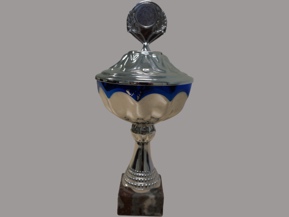 |
| **9 – TV Remote** | **10 – Thestral Figurine** | **11 – Power Strip** | **12 – Stuffed Spaghetti Monster** |
| 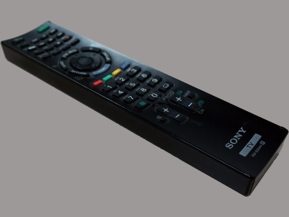 | 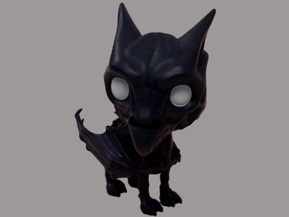 | 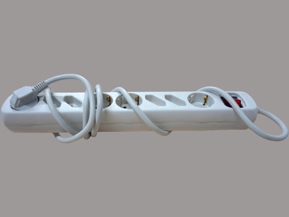 | 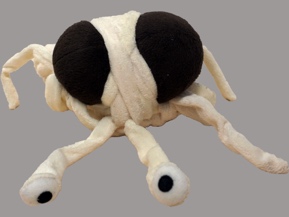 |
| **13 – Stuffed Squid** | **14 – Sunglasses** | **15 – Flowers in Vase** | **16 – Lunch Box Green** |
| 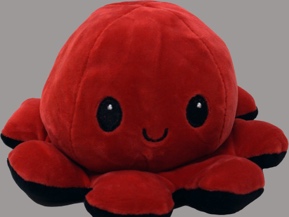 | 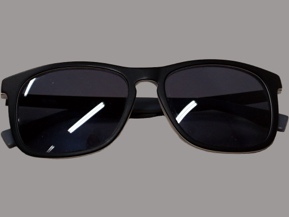 | 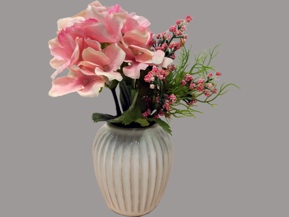 | 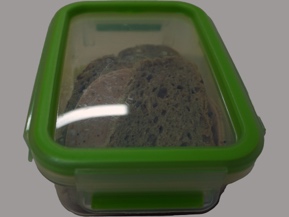 |
| **17 – Ice Cube Form** | **18 – Watering Can** | **19 – Mug Pink** | **20 – Tea Strainer** |
| 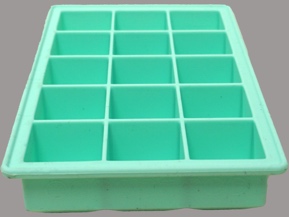 | 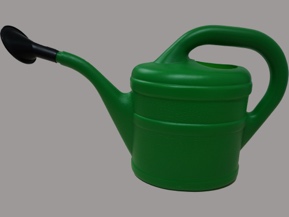 | 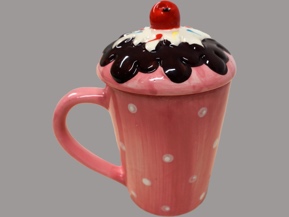 | 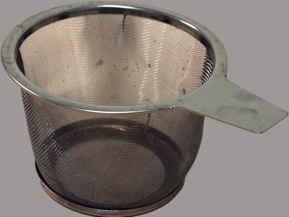 |
| **21 – Paintbox** | **22 – Bedroom Slipper** | **23 – Pencil Case pink** | **24 – Blue Box** |
| 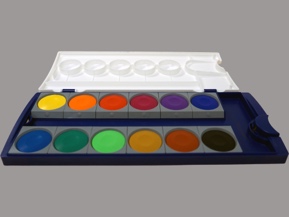 | 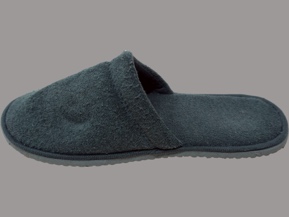 | 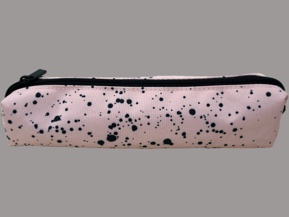 | 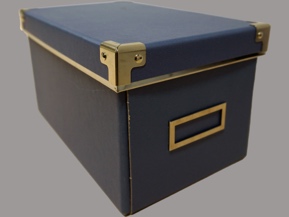 |

| **25 – Yoga Block** | **26 – Umbrella** | **27 – Folding Rule** | **28 – Dustpan Blue** |
| --- | --- | --- | --- |
| 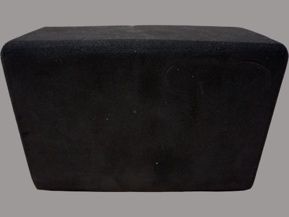 | 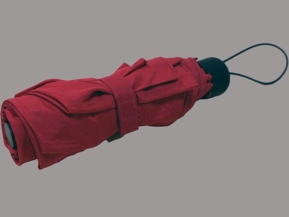 | 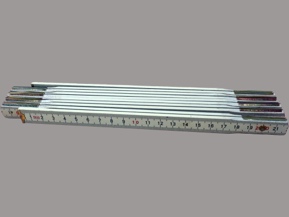 | 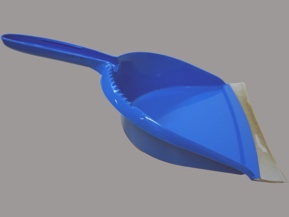 |
| **29 – Ship Figurine** | **30 – Chess Board** | **31 – Ballet Figurine** | **32 – Plastic Plant** |
| 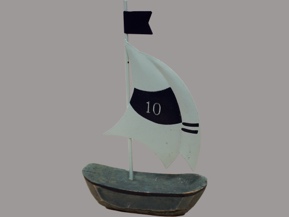 | 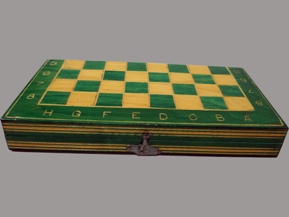 | 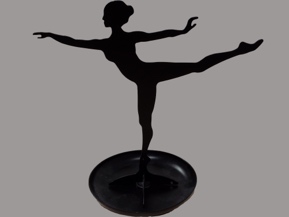 | 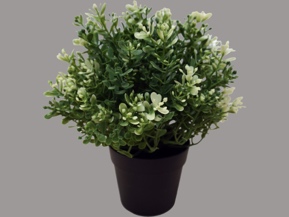 |
| **33 – Hair Brush** | **34 – Paper Bag** | **35 – Cotton Swab** | **36 –Tealight holder glass** |
| 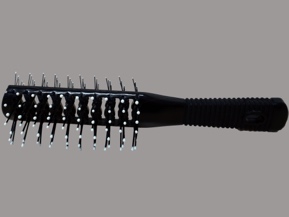 | 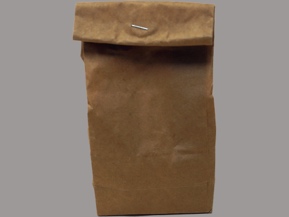 | 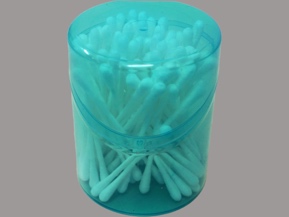 | 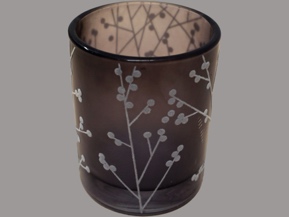 |
| **37 – Stuffed Flamingo** | **38 – Smartphone Holder** | **39 – Hawaiian lei necklace** | **40 – Pencil case colorful** |
| 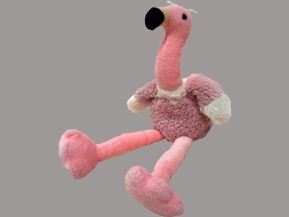 | 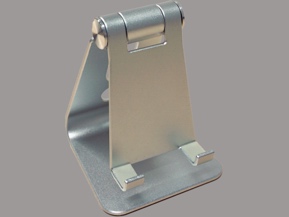 | 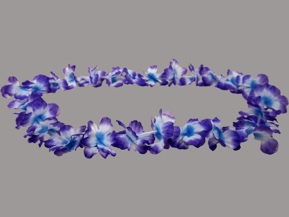 | 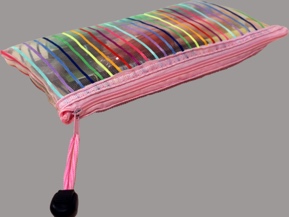 |
| **41 – Banana** | **42 – Boulder Shoe** | **43 – Jewelry Box** | **44 – Wood Log** |
| 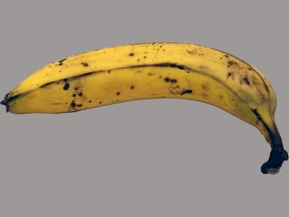 | 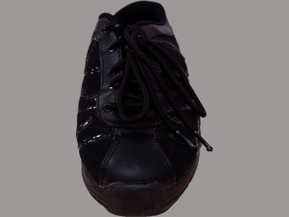 | 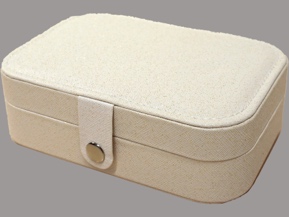 | 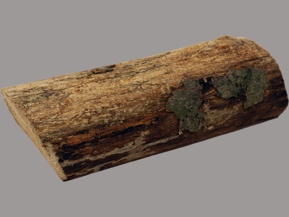 |
| **45 – Lunch Box Pink** | **46 – Camera battery** | **47 – Jar of Oats** | **48 – Headband** |
| 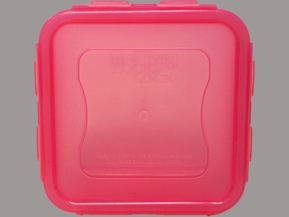 | 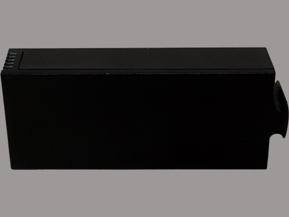 | 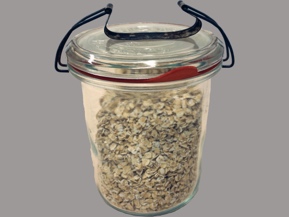 | 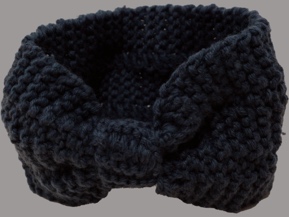 |

| **49 – Striped clay jug** | **50 – Dish Soap** | **51 – Pizza Cutter** | **52 – Recorder** |
| --- | --- | --- | --- |
| 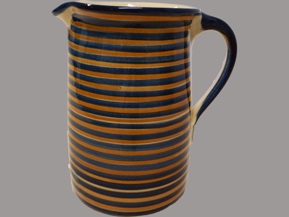 | 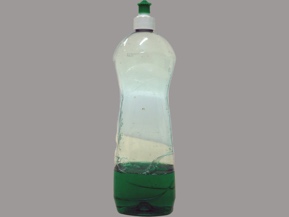 | 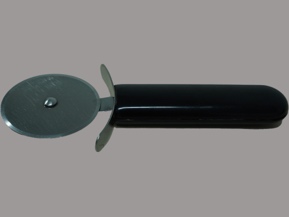 | 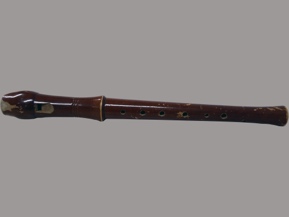 |
| **53 – Lighthouse** | **54 – Measuring instrument** | **55 – Rubber Duck** | **56 – Stamp Pad** |
| 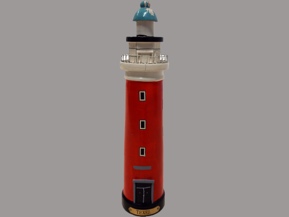 | 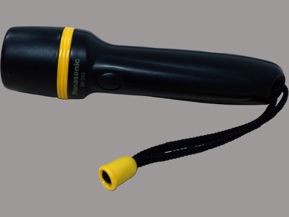 | 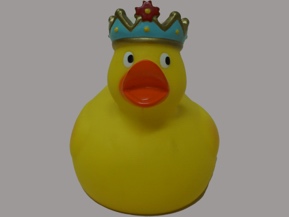 | 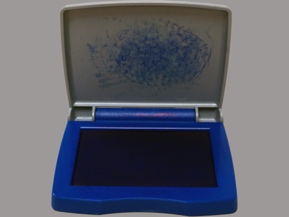 |
| **57 – Desk calendar** | **58 – Notepad** | **59 – Skipping rope** | **60 – Plastic Cone** |
| 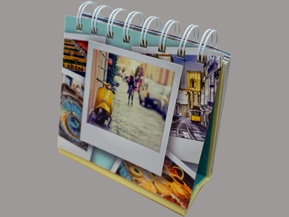 | 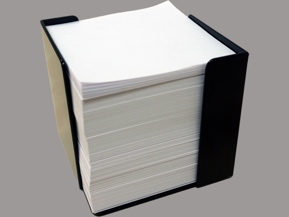 | 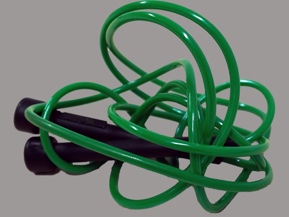 | 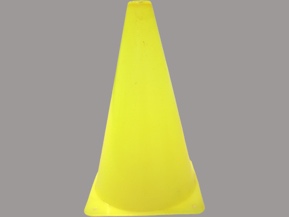 |

**Table S2**

*Results of the 2x2x4 repeated-measurements ANOVA (rmANOVA) with the within-subject factors “modality” (VR vs. PC), “target” (target vs. no target) and “stimulus type” (2 items vs. 3 items vs. 4 items vs. 5 items) for the response times and error rates & Results of the 2x4 repeated-measurements ANOVA (rmANOVA) with the within-subject factors “modality” (VR vs. PC) and “stimulus type” (2 items vs. 3 items vs. 4 items vs. 5 items) for parietal iABR*

|  | *df* | | *F* | | *p* | | *p*_corr_ | *η^2^* | *BF*_incl_ |
| --- | --- | --- | --- | --- | --- | --- | --- | --- | --- |
| **Response Times** | |  | |  | |  | | |  |
| Modality | 1,30 | | 4.79 | | .036* | | - | 0.14 | 1.4 |
| Target | 1,30 | | 8.85 | | .006* | | - | 0.23 | 3.7 |
| Setsize | 3,90 | | 69.95 | | <.001* | | - | 0.70 | 1.8x10^+14^ |
| Modality * Target | 3,30 | | 3.81 | | .060 | | - | 0.11 | 1.2 |
| Modality * Setsize | 2.1,62.4 | | 1.12 | | .344 | | .333 | 0.04 | 0.2 |
| Target * Setsize | 3,90 | | 0.89 | | .451 | | - | 0.03 | 0.2 |
| Modality * Target * Setsize | 3,90 | | 0.51 | | .676 | | - | 0.02 | 0.0 |
| **Error Rates** | |  | |  | |  | | |  |
| Modality | 1,30 | | 0.00 | | 1.000 | | - | 0.00 | 0.1 |
| Target | 1,30 | | 6.19 | | .019* | | - | 0.17 | 1.9 |
| Setsize | 3,90 | | 8.80 | | <.001* | | - | 0.23 | 56.9 |
| Modality * Target | 3,30 | | 0.42 | | .520 | | - | 0.01 | 0.1 |
| Modality * Setsize | 1,30 | | 2.18 | | .096 | | - | 0.07 | 0.2 |
| Target * Setsize | 3,90 | | 2.83 | | .043* | | - | 0.09 | 0.8 |
| Modality * Target * Setsize | 3,90 | | 1.22 | | .308 | | - | 0.04 | 0.0 |
| **Parietal iABR** | |  | |  | |  | | |  |
| Modality | 1,30 | | 13.57 | | <.001* | | - | 0.31 | 27.0 |
| Setsize | 3,90 | | 3.80 | | .013* | | - | 0.11 | 2.2 |
| Modality * Setsize | 3,90 | | 0.41 | | .747 | | - | 0.01 | 0.2 |

Notes: *α=.05, *p*_corr_ = Greenhouse-Geisser corrected p-value, *BF*_incl_ = Bayes Factor for factor inclusion. A *BF*_incl_ > 1 favors the inclusion of the factor in the ANOVA model, a *BF*_incl_ < 1 favors the exclusion of a factor.

**Table S3**

*Descriptive statistics (mean, standard deviation, confidence interval) for the response times [ms], error rates [no. of incorrect answers], and parietal iABR [*µV*] for all conditions*

|  | | *M* | *SD* | Confidence interval | |
| --- | --- | --- | --- | --- | --- |
|  | |  |  | Lower limit | Upper limit |
| **Response Times** | |  |  |  | |
| *PC - Target* | |  |  |  | |
| 2 items | | 720.98 | 136.0 | 671.1 | 770.9 |
| 3 items | | 805.13 | 143.2 | 752.6 | 857.6 |
| 4 items | | 820.50 | 153.4 | 764.2 | 876.8 |
| 5 items | | 835.35 | 128.9 | 788.1 | 882.6 |
| *PC – No Target* | |  |  |  |  |
| 2 items | | 750.21 | 133.2 | 701.3 | 799.1 |
| 3 items | | 799.66 | 127.5 | 752.9 | 846.4 |
| 4 items | | 846.16 | 152.4 | 790.3 | 902.1 |
| 5 items | | 842.47 | 130.4 | 794.7 | 890.3 |
| *VR - Target* | |  |  |  | |
| 2 items | | 685.76 | 121.4 | 641.2 | 730.3 |
| 3 items | | 738.50 | 104.1 | 700.3 | 776.7 |
| 4 items | | 770.08 | 124.9 | 724.3 | 815.9 |
| 5 items | | 802.52 | 127.9 | 755.6 | 849.4 |
| *VR – No Target* | |  |  |  |  |
| 2 items | | 724.45 | 117.5 | 681.4 | 767.6 |
| 3 items | | 776.32 | 134.6 | 726.9 | 825.7 |
| 4 items | | 815.92 | 122.4 | 771.0 | 860.8 |
| 5 items | | 832.31 | 102.3 | 794.8 | 869.8 |
| **Error Rates** | |  |  |  | |
| *PC - Target* | |  |  |  | |
| 2 items | | 1.68 | 2.3 | 0.8 | 2.5 |
| 3 items | | 2.58 | 2.6 | 1.6 | 3.6 |
| 4 items | | 2.32 | 2.2 | 1.5 | 3.3 |
| 5 items | | 2.42 | 2.5 | 1.5 | 3.1 |
| *PC – No Target* | |  |  |  |  |
| 2 items | | 1.23 | 1.6 | 0.6 | 1.8 |
| 3 items | | 1.16 | 1.7 | 0.5 | 1.8 |
| 4 items | | 1.48 | 1.7 | 0.8 | 2.1 |
| 5 items | | 1.39 | 1.8 | 0.7 | 2.0 |
| *VR - Target* | |  |  |  | |
| 2 items | | 1.77 | 2.0 | 1.0 | 2.5 |
| 3 items | | 2.03 | 2.4 | 1.2 | 2.9 |
| 4 items | | 2.71 | 2.2 | 1.9 | 3.5 |
| 5 items | | 2.10 | 1.9 | 1.4 | 2.8 |
| *VR – No Target* | |  |  |  |  |
| 2 items | | 1.06 | 1.4 | 0.6 | 1.6 |
| 3 items | | 0.90 | 1.3 | 0.4 | 1.4 |
| 4 items | | 1.84 | 2.0 | 1.1 | 2.6 |
| 5 items | | 1.84 | 1.7 | 1.2 | 2.5 |
| **Parietal iABR** | |  |  |  | |
| *PC* |  | |  |  | |
| 2 items | -0.59 | | 1.0 | -1.0 | -0.2 |
| 3 items | -0.49 | | 1.2 | -0.9 | -0.1 |
| 4 items | -0.42 | | 0.9 | -0.7 | -0.1 |
| 5 items | 0.03 | | 0.9 | -0.3 | 0.3 |
| *VR* |  | |  |  |  |
| 2 items | -1.17 | | 1.2 | -1.6 | -0.7 |
| 3 items | -1.03 | | 1.4 | -1.6 | -0.5 |
| 4 items | -1.06 | | 1.5 | -1.6 | -0.5 |
| 5 items | -0.75 | | 1.0 | -1.1 | -0.4 |

**Table S4**

*Pairwise comparison of the interaction effect “Target*Setsize” for the error rates*

|  | *df* | *T* | *p* (one-sided*)* | *p* (two-sided) | Cohen´s *d* | *BF*_10_ |
| --- | --- | --- | --- | --- | --- | --- |
| **Error Rates**  *Target vs. non-target* | | | | | | |
| 2 items | 30 | 1.82 | .039* | .079 | 0.3 | 0.8 |
| 3 items | 30 | 3.10 | .002** | .004** | 0.6 | 9.3 |
| 4 items | 30 | 2.21 | .017* | .035* | 0.4 | 1.6 |
| 5 items | 30 | 1.73 | .047* | .094 | 0.3 | 0.7 |

Notes: *significant for α=.05, **significant after Bonferroni-correction (α=.01), *BF*_10_ = Bayes Factor for H1 compared to H0. A *BF*_10_ > 1 favors the H1, while a *BF*_10_ < 1 favors the H0.

**
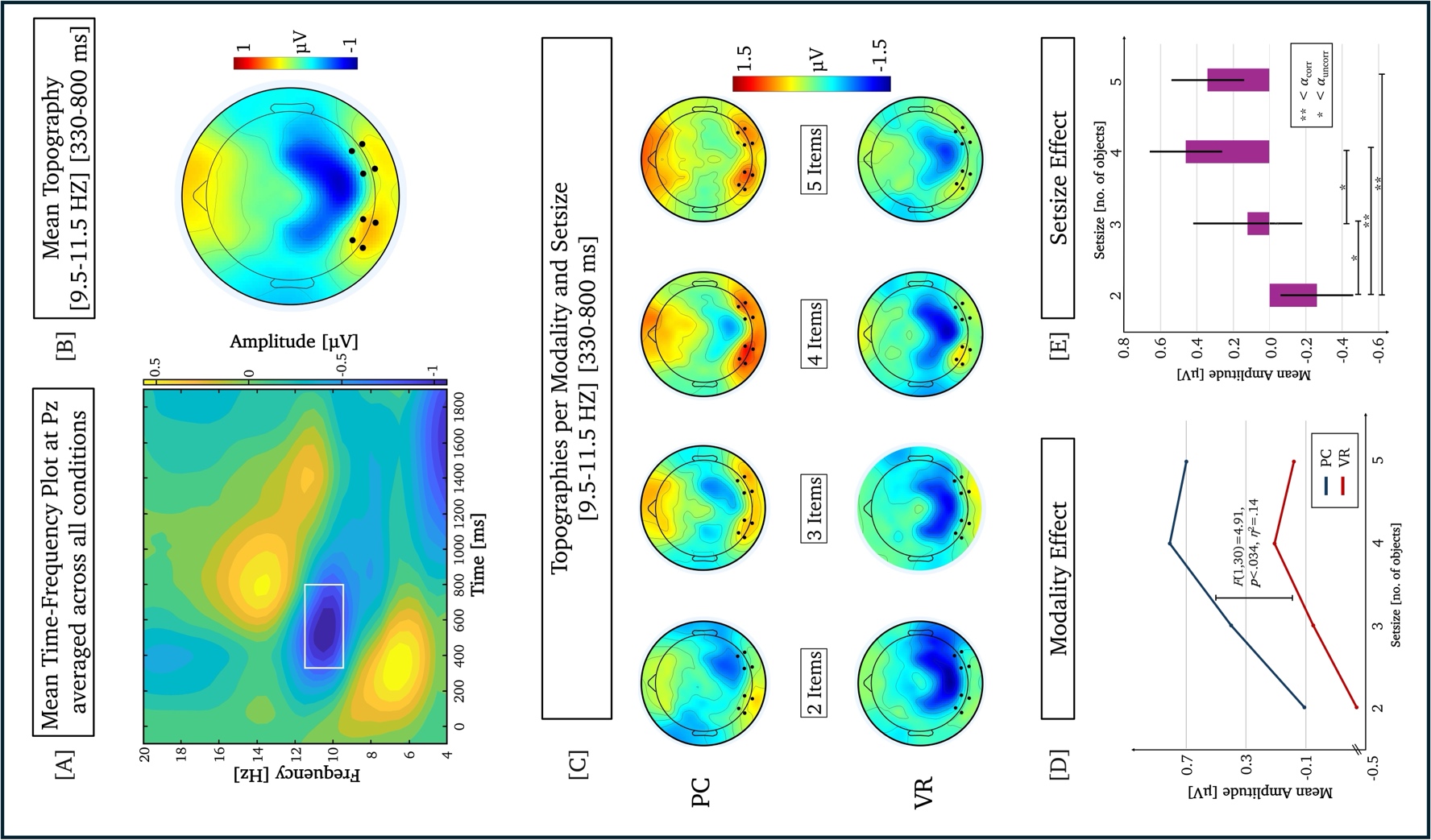
**

**Fig. S5**

[A] Mean Time-Frequency (TF) plot of the induced oscillatory signal at Pz averaged over all conditions. The iABR frequency range (9.5 – 11.5 Hz) and time window (330 – 800 ms) of interest is marked by a white rectangle. [B] Mean topographic distribution of the iABR within the time window of interest, averaged over all conditions. Electrodes selected for analysis are marked by white dots (O1, PO7, O2, PO8 and surrounding). [C] Topographic distribution of the iBBR per modality and setsize within the time window of interest. Electrodes selected for analysis are marked by black or white dots. [D] Mean iBBR amplitudes for PC and VR per setsize averaged across target and non-target trials (Per Modality). [E] Mean iBBR amplitudes setsize averaged across modality and target (Setsize Effect).

**
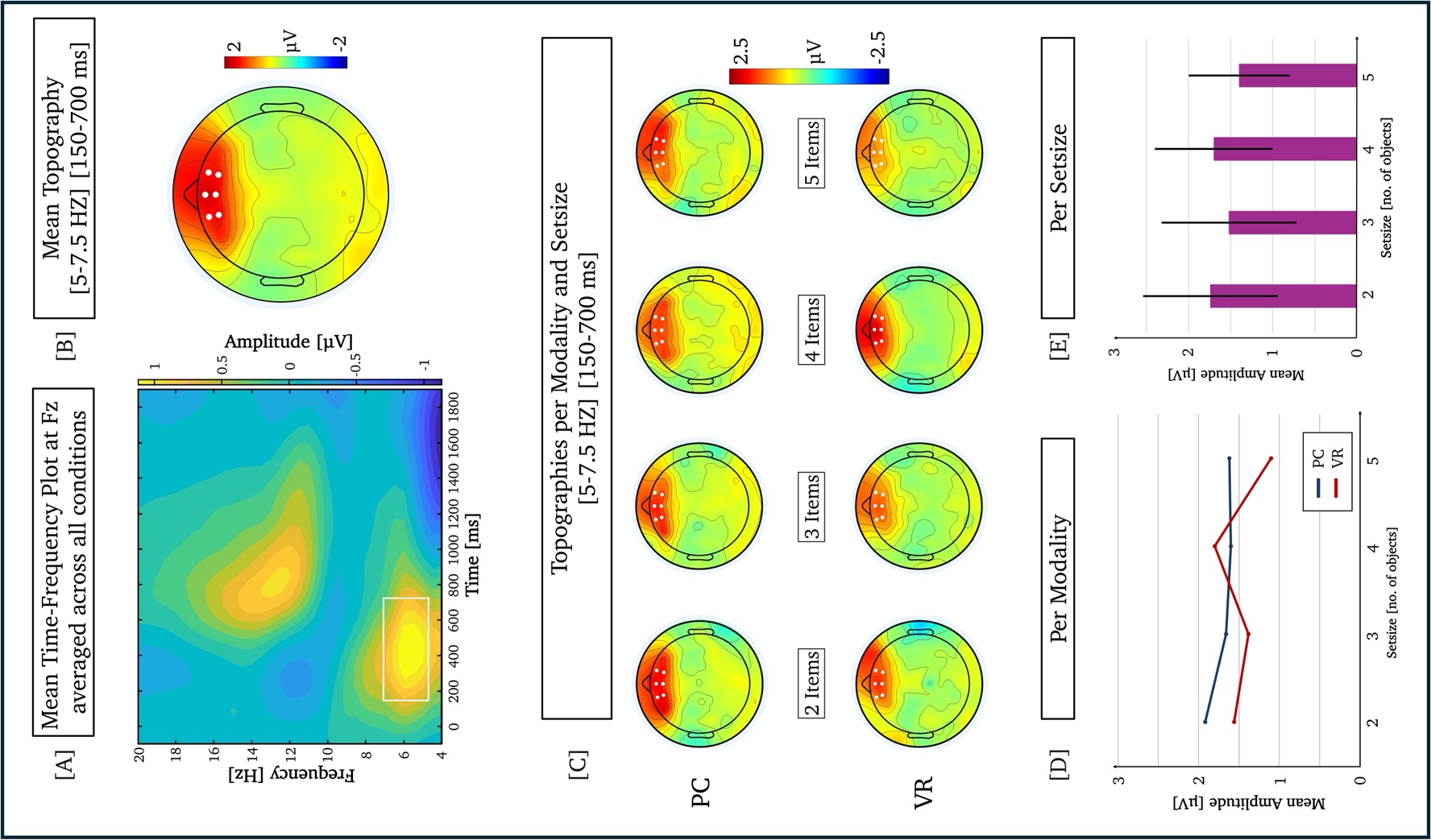
**

**Fig. S6**

[A] Mean Time-Frequency (TF) plot of the induced oscillatory signal at Fz averaged over all conditions. The iTBR frequency range (5 – 7.5 Hz) and time window (150 – 700 ms) of interest is marked by a white rectangle. [B] Mean topographic distribution of the iTBR within the time window of interest, averaged over all conditions. Electrodes selected for analysis are marked by white dots (AFz, AF3, AF4 and surrounding). [C] Topographic distribution of the iTBR per modality and setsize within the time window of interest. Electrodes selected for analysis are marked by black or white dots. [D] Mean iTBR amplitudes for PC and VR per setsize averaged across target and non-target trials (Per Modality). [E] Mean iTBR amplitudes setsize averaged across modality and target (Per Setsize).


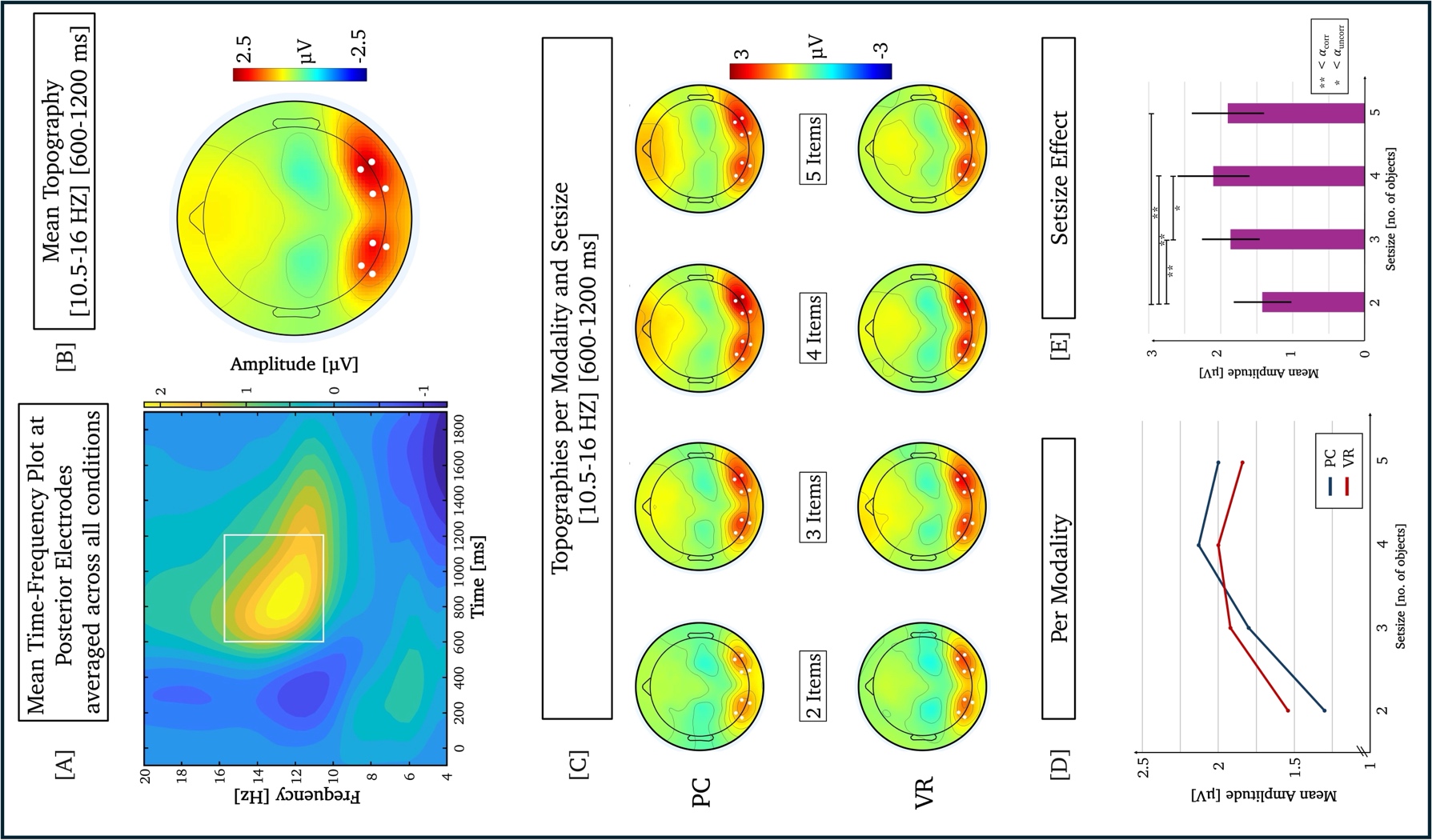


**Fig. S7**

[A] Mean Time-Frequency (TF) plot of the induced oscillatory signal at posterior electrodes averaged over all conditions. The iABR/iBBR frequency range (10.5 – 16 Hz) and time window (600 – 1200 ms) of interest is marked by a white rectangle. [B] Mean topographic distribution of the iABR/iBBR within the time window of interest, averaged over all conditions. Electrodes selected for analysis are marked by white dots (O1, PO7, O2, PO8 and surrounding). [C] Topographic distribution of the iABR/iBBR per modality and setsize within the time window of interest. Electrodes selected for analysis are marked by black or white dots. [D] Mean iABR/iBBR amplitudes for PC and VR per setsize averaged across target and non-target trials (Per Modality). [E] Mean iABR/iBBR amplitudes setsize averaged across modality and target (Setsize Effect).

**Table S8**

*Descriptive statistics (mean, standard deviation, confidence interval) for posterior iABR (330-800ms), frontal iTBR (150-700ms) and posterior iABR/iBBR (600-1200ms) [*µV*] for all conditions*

|  | *M* | *SD* | Confidence interval | |
| --- | --- | --- | --- | --- |
|  |  |  | Lower limit | Upper limit |
| **Posterior iABR (330-800ms)** | | | | |
| *PC* |  |  |  | |
| 2 items | -0.09 | 0.9 | -0.4 | 0.3 |
| 3 items | 0.40 | 1.3 | -0.1 | 0.9 |
| 4 items | 0.81 | 1.2 | 0.4 | 1.2 |
| 5 items | 0.70 | 1.4 | 0.2 | 1.2 |
| *VR* |  |  |  | |
| 2 items | -0.44 | 1.5 | -1.0 | 0.1 |
| 3 items | -0.15 | 2.4 | -1.0 | 0.7 |
| 4 items | 0.11 | 2.1 | -0.6 | 0.9 |
| 5 items | -0.02 | 1.9 | -0.7 | 0.7 |
| **Frontal iTBR (150-700ms)** | | | | |
| *PC* |  |  |  | |
| 2 items | 1.92 | 5.7 | -0.2 | 4.0 |
| 3 items | 1.66 | 5.4 | -0.3 | 3.6 |
| 4 items | 1.60 | 4.5 | 0.0 | 3.2 |
| 5 items | 1.62 | 4.4 | 0.0 | 3.2 |
| *VR* |  |  |  | |
| 2 items | 1.56 | 4.0 | 0.1 | 3.0 |
| 3 items | 1.38 | 3.6 | 0.1 | 2.7 |
| 4 items | 1.80 | 3.5 | 0.5 | 3.1 |
| 5 items | 1.10 | 2.9 | 0.0 | 2.2 |
| **Posterior iABR/iBBR (600-1200ms)** | | | | |
| *PC* |  |  |  | |
| 2 items | 1.30 | 1.8 | 0.7 | 1.9 |
| 3 items | 1.80 | 2.5 | 0.9 | 2.7 |
| 4 items | 2.13 | 2.2 | 1.3 | 2.9 |
| 5 items | 1.96 | 2.5 | 1.0 | 2.9 |
| *VR* |  |  |  | |
| 2 items | 1.54 | 2.6 | 0.6 | 2.5 |
| 3 items | 1.92 | 2.8 | 0.9 | 2.9 |
| 4 items | 2.02 | 3.1 | 0.9 | 3.1 |
| 5 items | 1.84 | 2.7 | 0.9 | 2.8 |

**Table S9**

*Results of the 2x4 repeated-measurements ANOVA (rmANOVA) with the within-subject factors “modality” (VR vs. PC) and “stimulus type” (2 items vs. 3 items vs. 4 items vs. 5 items) for posterior iABR (330-800ms), frontal iTBR (150-700ms) and posterior iABR/iBBR (600-1200ms)*

|  | *df* | *F* | *p* | *p*_corr_ | *η^2^* | *BF*_incl_ |
| --- | --- | --- | --- | --- | --- | --- |
| **Posterior iABR (330-800ms)** | | | | | | |
| Modality | 1,30 | 4.91 | .034* | - | 0.14 | 1.376 |
| Setsize | 3,90 | 6.51 | <.001* | - | 0.18 | 4.643 |
| Modality * Setsize | 2.4,71.3 | 0.31 | .821 | .774 | 0.01 | 0.146 |
| **Frontal iTBR (150-700ms)** | | | | | | |
| Modality | 1,30 | 0.43 | .519 | - | 0.01 | 0.204 |
| Setsize | 3,90 | 0.59 | .622 | - | 0.02 | 0.042 |
| Modality * Setsize | 3,90 | 0.62 | .602 | - | 0.02 | 0.005 |
| **Posterior iABR/iBBR (600-1200ms)** | | | | | | |
|  |  |  |  |  |  |  |
| Modality | 1,30 | 0.03 | .866 | - | 0.00 | 0.181 |
| Setsize | 3,90 | 7.15 | <.001* | - | 0.19 | 11.625 |
| Modality * Setsize | 3,90 | 0.61 | .612 | - | 0.02 | 0.076 |
|  |  |  |  |  |  |  |

Notes: *α=.05, *p*_corr_ = Greenhouse-Geisser corrected p-value, *BF*_incl_ = Bayes Factor for factor inclusion. A *BF*_incl_ > 1 favors the inclusion of the factor in the ANOVA model, a *BF*_incl_ < 1 favors the exclusion of a factor.

**Table S10**

*Pairwise comparison of the main factor “Setsize” for posterior iABR (330-800ms) and posterior iABR/iBBR (600-1200ms)*

|  |  | *t*(30) | *p* (one-sided*)* | *p* (two-sided) | Cohen´s *d* | *BF*_10_ |
| --- | --- | --- | --- | --- | --- | --- |
| **Posterior iABR (330-800ms)** | | | | | | |
| 2 vs. 3 items |  | -2.00 | .028* | .056 | -0.4 | 0.023 |
| 2 vs. 4 items |  | -4.15 | <.001** | <.001** | -0.7 | 2.588x10^-4^ |
| 2 vs. 5 items |  | -3.36 | .001** | .002** | -0.6 | 1.757x10^-8^ |
| 3 vs. 4 items |  | 0.00 | .026* | .051 | -0.4 | 0.022 |
| 3 vs. 5 items |  | 0.17 | .131 | .263 | -0.2 | 0.033 |
| 4 vs. 5 items |  | 0.82 | .209 | .418 | 0.1 | 0.035 |
| **Posterior iABR/iBBR (600-1200ms)** | | | | | | |
| 2 vs. 3 items |  | -3.20 | .002** | .003** | -0.6 | 5.834x10^-8^ |
| 2 vs. 4 items |  | -3.89 | <.001** | <.001** | -0.7 | 2.684x10^-7^ |
| 2 vs. 5 items |  | -2.74 | .005* | .010* | -0.6 | 4.447x10^-7^ |
| 3 vs. 4 items |  | -1.80 | .041* | .083 | -0.3 | 0.026 |
| 3 vs. 5 items |  | -0.30 | .382 | .763 | -0.1 | 0.036 |
| 4 vs. 5 items |  | 1-37 | .090 | .180 | 0.2 | 0.031 |

Notes: *significant for α=.05, **significant after Bonferroni-correction (α=.008), *BF*_10_ = Bayes Factor for H1 compared to H0. A *BF*_10_ > 1 favors the H1, while a *BF*_10_ < 1 favors the H0.
